# Antagonistic Effects of Point Mutations on Charge Recombination and a New View of Primary Charge Separation in Photosynthetic Proteins

**DOI:** 10.1021/acs.jpcb.1c03978

**Published:** 2021-07-30

**Authors:** K. Dubas, S. Szewczyk, R. Białek, G. Burdziński, M. R. Jones, K. Gibasiewicz

**Affiliations:** † Faculty of Physics, Adam Mickiewicz University, ul. Uniwersytetu Poznanskiego 2, 61-614 Poznań, Poland; ‡ School of Biochemistry, Medical Sciences Building, University of Bristol, University Walk, Bristol, BS8 1TD, U.K.; § Department of Optometry, Poznan University of Medical Sciences, ul. Rokietnicka 5d, 60-806 Poznań, Poland

## Abstract

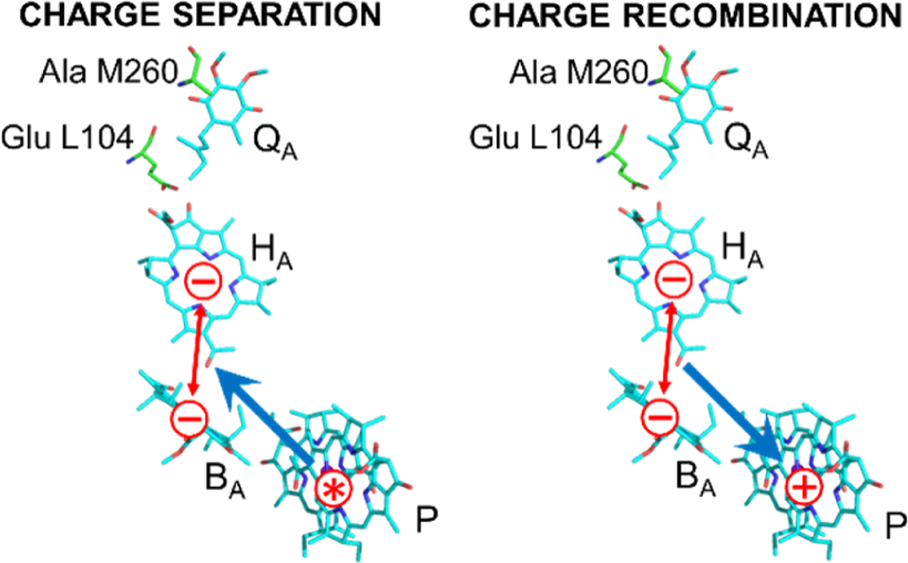

Light-induced electron-transfer reactions were investigated in wild-type and three mutant *Rhodobacter sphaeroides* reaction centers with the secondary electron acceptor (ubiquinone Q_A_) either removed or permanently reduced. Under such conditions, charge separation between the primary electron donor (bacteriochlorophyll dimer, P) and the electron acceptor (bacteriopheophytin, H_A_) was followed by P^+^H_A_^–^ → PH_A_ charge recombination. Two reaction centers were used that had different single amino-acid mutations that brought about either a 3-fold acceleration in charge recombination compared to that in the wild-type protein, or a 3-fold deceleration. In a third mutant in which the two single amino-acid mutations were combined, charge recombination was similar to that in the wild type. In all cases, data from transient absorption measurements were analyzed using similar models. The modeling included the energetic relaxation of the charge-separated states caused by protein dynamics and evidenced the appearance of an intermediate charge-separated state, P^+^B_A_^–^, with B_A_ being the bacteriochlorophyll located between P and H_A_. In all cases, mixing of the states P^+^B_A_^–^ and P^+^H_A_^–^ was observed and explained in terms of electron delocalization over B_A_ and H_A_. This delocalization, together with picosecond protein relaxation, underlies a new view of primary charge separation in photosynthesis.

## Introduction

Studies of light-induced electron-transfer (ET) reactions in photosynthetic proteins are important because similar ET processes occur commonly in many other proteins and play vital functional roles. Moreover, the transformation of an excited electronic state into a charge-separated state is a crucial reaction in many artificial photovoltaic devices; so, understanding this natural process may provide important guidelines on how to construct such devices for efficient energy conversion.

Photosynthetic reaction centers (RCs) are the pigment–protein complexes in which the energy of absorbed light is transformed into that of charge-separated states.^1−4^ The primary charge separation in RCs, the initial step of ET, occurs between a (bacterio)chlorophyll species, denoted P, and a nearby (bacterio)chlorin acceptor within a few picoseconds.^5−7^

**Figure 1:**
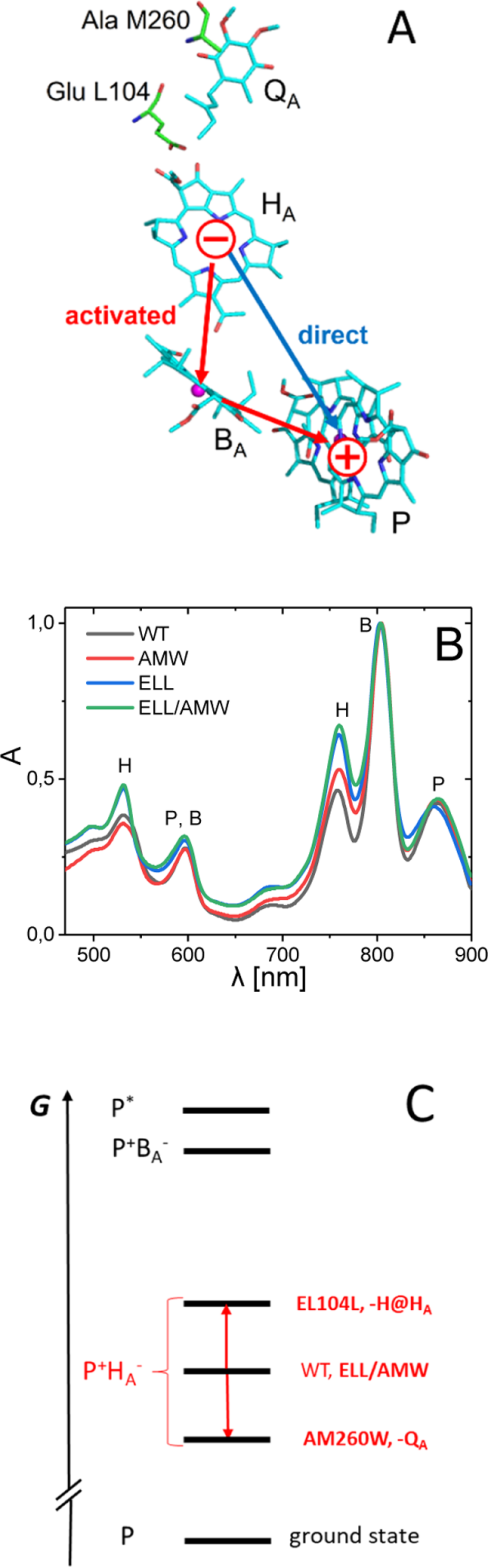
(A) Arrangement of the ET cofactors in the WT *Rhodobacter sphaeroides* RC. Amino acids replaced in the mutant strains as well as postulated direct and activated back-ET routes are also shown. (B) Ground-state absorption spectrum of the WT and mutant RCs normalized at ∼804 nm. (C) Comparison of the expected effects of the single (ELL and AMW) and double (ELL/AMW) point mutations on the relative free energy level of the state P^+^H_A_^–^.

One of the most widely studied RCs is that from the purple bacterium *Rhodobacter (Rba.) sphaeroides* (Figure 1A,B).^6−9^ Three redox centers embedded in the protein are involved in the primary charge separation reaction, namely, the dimeric BChl primary electron donor (P), a monomeric BChl, B_A_, and a bacteriopheophytin (BPhe), H_A_. The first relatively easily detectable charge-separated state, P^+^H_A_^–^, includes only two of these centres.^5^ However, more detailed studies have shown that the B_A_ BChl, located between P and H_A_, is involved in the formation of a primary charge-separated state either as a virtual electron carrier connecting P* to H_A_ in the superexchange mechanism^10^ or, more likely, as a real intermediate electron carrier, according to the following sequential biphasic reaction^11,12^
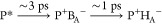
1

The possibility that both of these sequential reactions are reversible has been deduced from the detailed global and target analyses of transient absorption data.^13,14^

Following the formation of the state P^+^H_A_^–^, the next forward ET step, completed within ∼200 ps, takes place from H_A_^–^ to the ubiquinone Q_A_.^15−17^ This forward reaction competes with back-ET to P^+^,^18^ and this charge recombination reaction may be written in a simplified way as
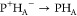
2

Because this charge recombination reaction occurs on a ∼10 ns time scale,^19−25^ it is rather difficult to observe it in an “open” RC, that is, an RC in which a forward ET to Q_A_ is possible and dominates because it is 2 orders of magnitude faster than recombination. A way to have access to this charge recombination reaction is to block the forward ET, either by the removal of Q_A_ chemically^20,25^ or genetically^27,28^ or by its permanent reduction to Q_A_^–^.^20−22,26^ The latter method introduces an electric field inside the protein which affects both the primary charge separation^26,29,30^ and the charge recombination.^31^

Similar to primary charge separation, studies of P^+^H_A_^–^ → PH_A_ charge recombination have demonstrated that it involves an intermediate state, P^+^B_A_^–^.^26,32^ The engagement of P^+^B_A_^–^, together with the dynamic relaxation of the protein triggered by the appearance of the charge-separated states leading to a dynamic increase of the free energy gap between the states P^+^H_A_^–^ and P^+^B_A_^–^, was found to be responsible for multiexponential charge recombination.^33−35^ Similar models have been used to explain variations in the charge recombination dynamics in a range of RCs with mutations^33,35^ and over a range of temperatures.^34^

The dynamics of proteins span many orders of magnitude from picoseconds to seconds and beyond.^36^ Their influence on ET in the RC has been postulated many times,^37−41^ but their experimental dissection from the complex ET kinetics is a real challenge that is not often undertaken.^42−44^ Perhaps, the most impressive attempt was a demonstration that in a range of mutated RCs with manipulated interactions between P and B_A_, on the one hand, and the protein environment, on the other hand, and thus with diverse P* decay kinetics (due to the primary charge separation), the kinetics of the protein, as monitored by the transient absorption signal from tryptophan residues at ∼280 nm, was mutation-independent.^42^ This apparent discrepancy was reconciled in the frame of a reaction-diffusion model, indicating that the protein dynamics actively trigger the primary charge separation rather than passively react to it. The detected kinetics of the protein occurred with 3, 10, and 190 ps lifetimes. A protein dynamics occurring on a 100 ps time scale was also proposed to prevent the P^+^H_A_^–^ → PH_A_ charge recombination in open RCs.^35^ Furthermore, the protein dynamics was hypothesized to affect the ET from H_A_^–^ to Q_A_.^44^ These experimental investigations were supported by theoretical approaches that considered protein dynamics in RCs on a time scale of up to 500 ps.^45−47^

In this contribution, we extend our previous kinetic model of primary charge separation and charge recombination in closed RCs, that is, in RCs with a blocked ET from H_A_^–^ to Q_A_,^32,48^ based on high-quality transient absorption data and their detailed global and target analyses. The data were collected for wild-type (WT) and three mutant RCs with blocked ET to Q_A_. Two of these RCs, ELL and AMW, have been reported previously in literature (see the Materials and Methods section for details). The ELL RC is characterized by a ∼3-fold acceleration in charge recombination compared to that of the WT RC. Contrastingly, the AMW RC, which is devoid of the Q_A_ ubiquinone secondary electron acceptor, shows a ∼3-fold slower charge recombination compared to that of the WT RC. The opposing effects of these mutations on the kinetics of charge recombination have been explained by their opposing influences on the free energy gap between the states P^+^H_A_^–^ and P^+^B_A_^–^ (Figure 1C) and thus different degrees of access to the fast, activated charge recombination pathway (Figure 1A).^33^ A third RC with both these mutations, denoted ELL/AMW, was newly constructed in this work in order to test the hypothesis that the opposing effects of the single mutations will cancel each other despite the different nature of the block of the forward ET from H_A_ (i.e., Q_A_ reduction vs. its absence). Additionally, our thorough kinetic analysis resulted in a new perspective on the primary charge separation reaction (eq 1), which appears to be coupled with a fast protein relaxation on the time scale of a few picoseconds. On the other hand, a slower protein relaxation phase occurring on the ∼500 ps to 2 ns time scale is responsible for the observed fast phase of the charge recombination. Thus, the protein dynamics appear to control ET reactions in RCs occurring on very different time scales ranging at least from picoseconds to nanoseconds.

## Materials and Methods

### Biological Materials

Purified RCs were prepared according to procedures described earlier.^33,49−51^ In addition to WT RCs, the following mutant complexes were studied: ELL (Glu L104 replaced by Leu), AMW (Ala M260 replaced by Trp), and ELL/AMW (a combination of both single mutations; see Figure 1A for the location of the replaced amino acids). Mutation AMW results in the exclusion of the Q_A_ ubiquinone from its binding site.^27,28,43^ Mutation ELL removes a hydrogen bond interaction with the H_A_ BPhe,^52^ thus increasing the free energy level of the state P^+^H_A_^–^.^33,53^ The double mutation is expected to combine these two effects.

For the transient absorption experiments, purified RCs were diluted in 15 mM Tris buffer (pH 8.0), containing 0.025% LDAO (*N*,*N*-dimethyldodecylamine-*N*-oxide). The final optical densities (OD_803nm,1cm_) of the samples were 0.3–0.4. During the experiment, the RC solution was housed in a stirred quartz cell (2 mm path length). In order to close RCs, normal ET from H_A_^–^ to Q_A_ was blocked either by the absence of Q_A_ (caused by the AMW mutation) or by the prereduction of Q_A_ to Q_A_^–^ (in the WT and ELL RCs). To achieve Q_A_ reduction, the samples were illuminated with a continuous background white halogen light (∼1 mW/cm^2^) in the presence of 10 mM of the external electron donor sodium ascorbate. Under this condition, low-intensity background illumination creates the state P^+^Q_A_^–^ and ascorbate re-reduces P^+^ to P (Gibasiewicz et al. 2009),^32^ resulting in the state PQ_A_^–^.

### Transient Absorption Measurements and Data Analysis

Femtosecond UV–vis–NIR transient absorption spectra were collected using a Helios transient absorption setup (Ultrafast Systems).^54^ The excitation beam was generated by a short-pulse titanium–sapphire oscillator (Mai-Tai, Spectra Physics, 70 fs), followed by a high-energy titanium–sapphire regenerative amplifier (Spitfire Ace, Spectra Physics, 100 fs, 1 kHz). The train of pulses exciting the sample was reduced to a 0.5 kHz repetition rate using a chopper. The 803 nm beam was split to form two beams: (1) pump (λ_exc_ = 803 nm) and (2) white light continuum probe pulses generated in a sapphire plate (440–780 nm).^54^ The remaining 803 nm photons in the probe pulse were filtered out by a BG 38 cutoff filter placed before the sample to avoid the additional excitation of the sample. The instrument response function was approximately 200 fs wide. The pump pulse energy was approximately 1 μJ at the sample position and this relatively high intensity did not introduce any unwanted nonlinear effects. All the experiments were performed at room temperature. The data were acquired in a ∼3 ns time window. For each delay time *t*, the Δ*A*(*t*) was calculated from recorded 1000 probe spectra (500 spectra with the pump and 500 spectra without the pump). The whole kinetics were collected twice, once with gradually increasing delay times between the pump and the probe and after that with gradually decreasing times, in order to ensure there were no systematic changes in the samples during the experiments. The 3 ns width of the experimental time window was smaller than the values of the slowest lifetimes obtained from fitting the experimental results. For this reason the nanosecond components reported below should be treated with caution as very approximate estimation of the slow charge recombination kinetics.

The results were corrected for the spectral chirp of the white-light continuum using SurfaceXplorer software (Ultrafast Systems). Glotaran software was used to perform global and target analyses.^55^ Steady-state absorption spectra were collected using a Hitachi-1900 UV–vis spectrophotometer.

## Results and Discussion

### Global Analysis

**Figure 2:**
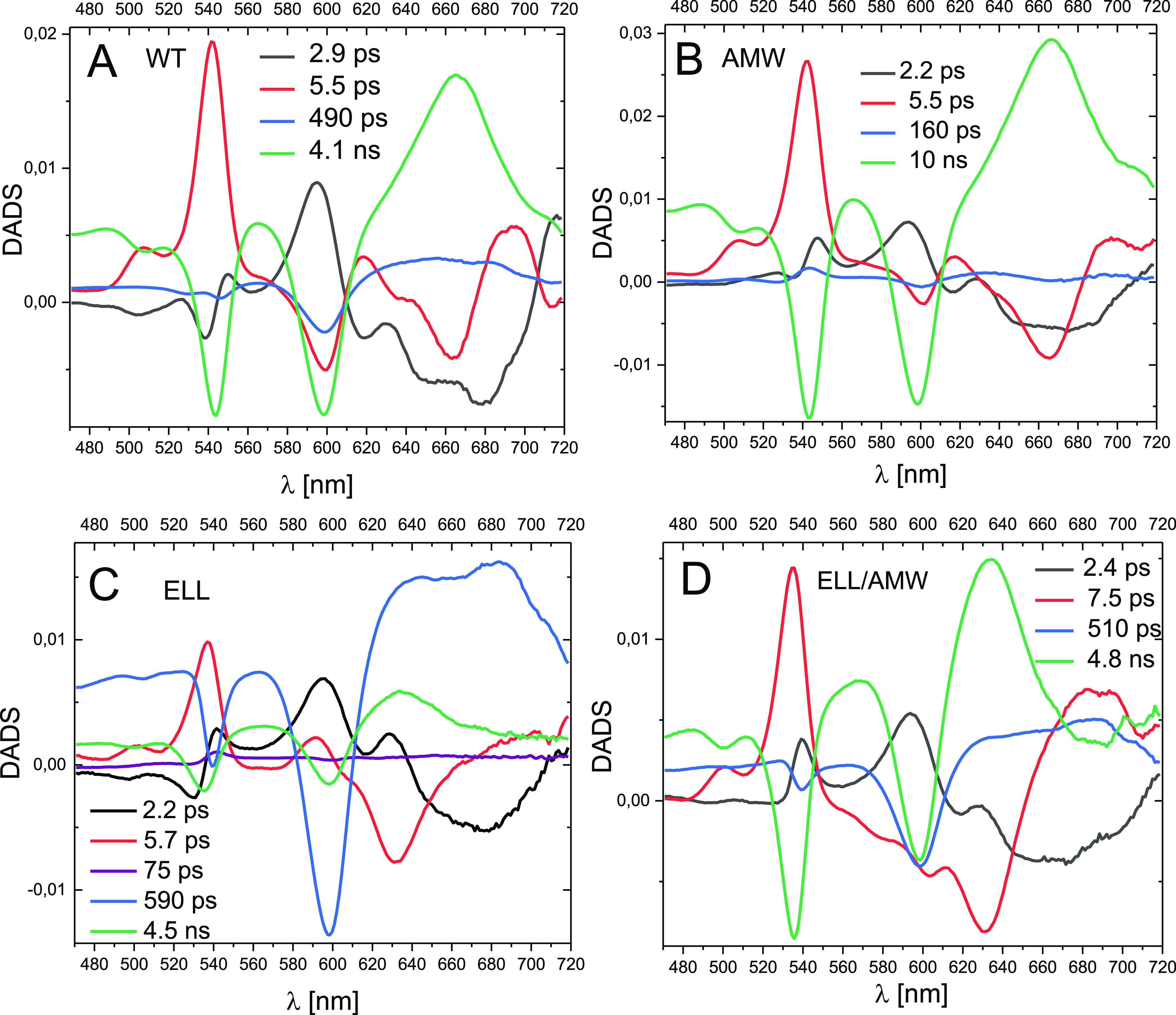
DADS for the WT and mutant RCs.

Femtosecond time-resolved absorption changes between 470 and 720 nm, triggered by the 803 nm to 100 fs excitation flashes, were recorded over a ∼3 ns time window for the WT RC and the three mutant complexes. Figure 2 presents decay-associated difference spectra (DADS) from a global analysis of these data. An appearance of photobleaching (PB) and excited-state absorption (ESA) is presented as positive and negative DADS, respectively, whereas the polarities of the DADS depicting decay of PB and ESA are inverse. The two fastest sub-picosecond components (of ∼150 and ∼300 fs lifetimes), depicting energy transfer from initially excited B* to P^56−60^ with a possible minor contribution of the direct charge separation from the state B*,^61,62^ were obtained for all the RCs but are not of primary interest in this paper and therefore are neither shown nor discussed in detail. All four data sets were characterized by the four remaining components with lifetimes ranging from ∼2 ps to ∼10 ns (Figure 2). The corresponding DADS in each of these data sets were of roughly similar lifetime, shape, and origin but had different relative contributions.

**Figure 3:**
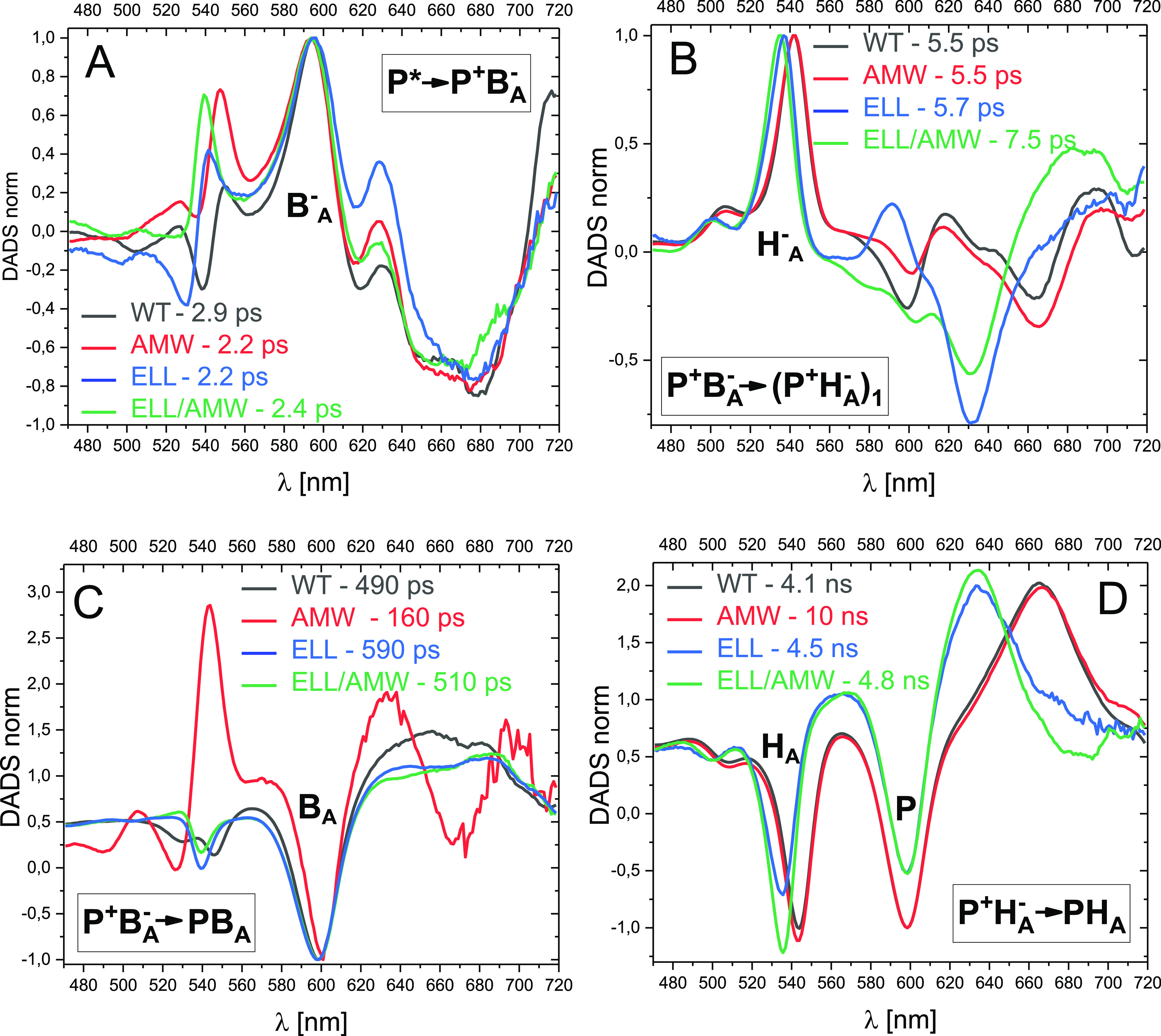
Comparison of the line shapes of normalized DADS obtained for the four RCs. (A) ∼2.5 ps DADS normalized at ∼595 nm; (B) ∼6 ps DADS normalized at ∼540 nm; (C) hundreds of picoseconds DADS normalized at ∼600 nm; and (D) nanosecond DADS normalized in such a way that the depth of the P PB band at 598 nm relative to the signal at 565 nm was the same for all spectra. (A–D)—the labels in the black rectangles indicate the reactions dominating the respective kinetic phases.

The similarity in the line shape of the corresponding DADS for the four RCs is demonstrated in Figure 3. The main features of the DADS of the component with a 2.2–2.9 ps lifetime (Figure 3A) were a positive band at ∼595 nm and a broad negative band with a relatively flat minimum between 650 and 690 nm. Both these features are clearly ascribable to the formation of the B_A_^–^ anion due to ET from P* to B_A_: the band at ∼595 nm is due to the appearance of PB of the B_A_ Q_x_ transition band, whereas the trough at 650–690 nm is due to the appearance of the transient absorption of B_A_^–^.^63^ The features around ∼540 nm may be due to a mixture of an electrochromic shift of H_A_ upon the formation of B_A_^–^ and formation of a certain amount of H_A_^–^ simultaneous with the formation of B_A_^–^.

The DADS of the component with a 5.5–7.5 ps lifetime (Figure 3B) were also dominated by two features, which this time were ascribable to the formation of the H_A_^–^ anion. These were a positive band peaking, depending on the sample, between 535 and 541 nm and a negative band at ∼665 nm (WT and AMW) or at ∼631 nm (ELL and ELL/AMW). The origin of these two features is analogous to that of the corresponding features described above for the B_A_^–^ formation: the positive band is due to the appearance of PB of the H_A_ Q_x_ band and the negative one to the appearance of the absorption of H_A_^–^ .^63^ Interestingly, the position of the negative band was strongly blue-shifted from 665 nm in the WT and AMW RCs to 631 nm in the ELL and ELL/AMW RCs. This effect, together with a smaller diversity in the 535–541 nm band position, is a result of the breaking of a hydrogen bond between glutamate 104 of the L sub-unit and the H_A_ BPhe in the ELL and ELL/AMW mutants and has been reported in an earlier study.^48^ It is worth noting that the sharp shapes of the transient absorption bands of H_A_^–^ at 665/631 nm are clearly different from the analogous flat broad bands of B_A_^–^ at 650–690 nm (Figure 3A). The two main features at 535–541 and 665/631 nm are accompanied by smaller and more difficult to explain changes in the ∼600 nm region. In the spectra of the WT, AMW, and ELL/AMW RCs, there is a negative band in this region. This could be due to the decay of B_A_^–^ PB caused by the ET from B_A_^–^ to H_A_. Conversely, in spectra of the ELL RC, the band at ∼600 nm is positive. This indicates that, on the 5.7 ps time scale, some formation of B_A_^–^ still occurs in the ELL RCs in parallel with the formation of H_A_^–^. Apparently, the 5.5–7.5 ps DADS, similarly to the 2.2–2.9 ps DADS, depict a mixed contribution from at least two processes. A non-trivial novelty of the presented global analysis was the clear resolution of the ∼2.5 and ∼6 ps processes, which previously were lumped together within a single kinetic process.^32,48^

It is also notable to compare the relative contributions of the 2.2–2.9 ps DADS (Figure 2, black spectra) and the 5.5–7.5 ps DADS in different samples (Figure 2, red spectra). The extreme cases are AMW, for which the B_A_^–^ PB formation band at ∼600 nm is the smallest, and ELL, for which the B_A_^–^ PB formation band is the largest.

The third common component had a lifetime of hundreds of picoseconds (Figure 3C), ranging from 490 to 590 ps for the WT, ELL, and ELL/AMW RCs. For WT and ELL RCs, similar kinetic components were resolved previously in transient absorption experiments performed and analyzed over a 100 ns experimental time window.^33^ For these three RCs, the main features of this third component were a negative band at 598 nm and a broad flat positive band between 620 and 700 nm. These features resemble inverted spectra from Figure 3A assigned to the formation of B_A_^–^ and thus are assigned partly to the decay of B_A_^–^ caused by P^+^B_A_^–^ → PB_A_ recombination. Due to this assignment, a part of the 598 nm band has to be ascribed to the decay of P^+^ PB. Importantly, the 490–590 ps DADS were additionally contributed to by negative bands peaking at 539 nm (ELL and ELL/AMW) or 546 nm (WT). These bands reveal the simultaneous decay of a fraction of H_A_^–^ caused by an equilibrium established between the two states: P^+^H_A_^–^ ↔ P^+^B_A_^–^. Because the 539–546 nm signal from H_A_^–^ is ∼3 times smaller than the ∼598 nm signal from B_A_^–^/P^+^, we estimate that the states P^+^H_A_^–^ and P^+^B_A_^–^ to be roughly isoenergetic on the time scale of a few hundreds of picoseconds. This estimation originates from a rough assumption that the differential extinction coefficient for H_A_^–^/H_A_ at ∼540 nm is the same as those for B_A_^–^/B_A_ and P^+^/P at ∼600 nm (see also DADS in Figure 3D).^63^ Under such an assumption, one-third of the ∼600 nm band is due to the decay of B_A_^–^ PB, one-third is due to P^+^ from the state P^+^B_A_^–^, and one-third is due to P^+^ from the state P^+^H_A_^–^. Thus, similar total contributions of P^+^B_A_^–^ and P^+^H_A_^–^ PB (at ∼540 nm and ∼600 nm) reveal the isoenergetic character of these two states: the same amount of the oscillator strength from P^+^B_A_^–^ and P^+^H_A_^–^ is lost within ∼500 ps. This issue is further commented below when discussing the shape of the “P^+^B_A_^–^” species-associated difference spectra (SADS).

The third component of the AMW RC had a shorter 160 ps lifetime, with a partially differently shaped DADS compared to the equivalent 490–590 ps DADS of the other RCs (Figure 3C). A negative band at ∼600 nm is accompanied by a positive band at 543 nm of similar amplitude. This indicates that the process underlying the 160 ps DADS was a slow ET from B_A_^–^ to H_A_. This assignment is further confirmed by the shape of this DADS between 620 and 720 nm, which looks like a wide positive band (due to the decay of transient absorption of B_A_^–^) interrupted by a sharper negative band at ∼670 nm (due to the formation of transient absorption of H_A_^–^). The shape of this DADS resembles that of the 5.5 ps DADS, except for a higher contribution of the B_A_^–^ decay features in the case of the former (see Figure S1). However, considering that the 160 ps DADs are very small, this difference may be due to some inaccuracy of the measurement (comparing the respective DADS in Figure 2B).

The DADS of the fourth, slowest component with a 4.1–10 ns lifetime showed features easily ascribable to the P^+^H_A_^–^ → PH_A_ recombination (Figure 3D). The shapes of these DADS were particularly similar for the WT and AMW RCs. For these, the negative signal at 598 nm is due to the P ground-state absorption recovery (decay of the Q_x_ band PB caused by transient formation of P^+^ cation), whereas the negative band at 544 nm is due to a similar recovery of the H_A_ Q_x_ band. These features are complemented by positive bands at ∼665 nm showing the decay of transient absorption of H_A_^–^ anions (compare this band to the respective negative bands in Figure 3B, depicting the formation of H_A_^–^). The equivalent DADS for the ELL and ELL/AMW RCs had differences that were attributable to the ELL mutation. In a similar fashion to the 5.5–7.5 ps DADS assigned mostly to the formation of the state P^+^H_A_^–^, both the PB band at ∼535 nm and transient absorption bands at 634 nm were blue-shifted compared to the bands at ∼545 and 665 nm, respectively, in the WT and AMW RCs. Generally, the shapes of the 4.5–4.8 ns DADS for the ELL and ELL/AMW RCs are very similar to each other except for a somewhat larger H_A_ PB band at ∼535 nm for the ELL/AMW RC.

The amplitudes of the 490–590 ps DADS, relative to those of the slowest 4.1–4.8 ns DADS, were very much different in the three RCs (Figure 2). For the ELL RC, the amplitude of the 490 ps DADS was, at all wavelengths, a few times larger than that of the 4.5 ns DADS, whereas in the WT and ELL/AMW RCs an opposite effect was observed, with the amplitudes of the 4.1/4.8 ns DADS being a few times larger than those of the 490/510 ps DADS. Thus, overall P^+^H_A_^–^ → PH_A_ recombination in the ELL RC was dominated by the ∼0.5 ns component, whereas in the WT and ELL/AMW RCs, the 4.1/4.8 ns recombination dominated. On the other hand, in the AMW RC, the P^+^H_A_^–^ → PH_A_ recombination occurred exclusively within ∼10 ns lifetime.

**Figure 4:**
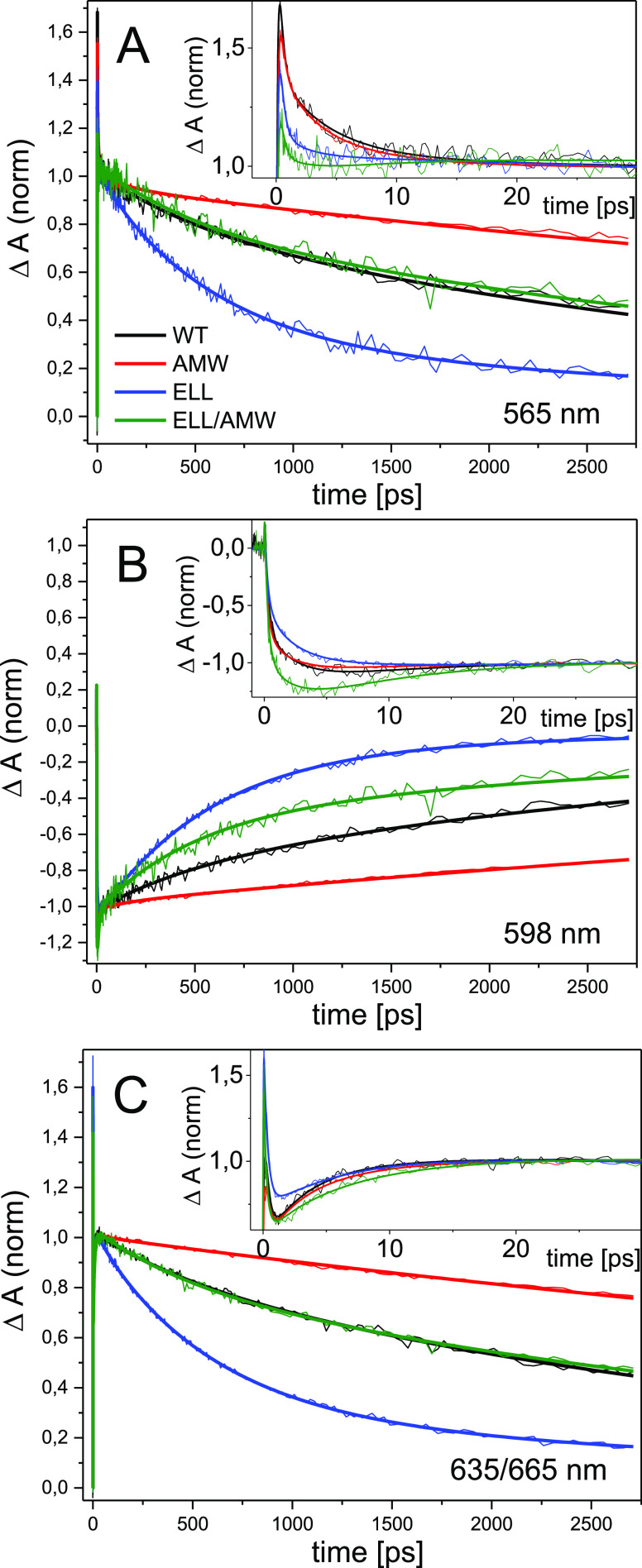
Comparisons of the ET kinetics at three selected wavelengths in the four RCs, normalized at ∼30 ps after excitation: (A) 565; (B) 598; and (C) 635 nm (ELL, ELL/AMW)/665 nm (WT and AMW). Insets: respective kinetics in 30 ps time windows. Fits are from the global analysis presented in Figure 2.

A comparison of the kinetics at three selected wavelengths, demonstrating these differences in the P^+^H_A_^–^ → PH_A_ recombination dynamics, is presented in Figure 4. The accelerated kinetics in the ELL RC and the decelerated kinetics in the AMW RC, relative to the WT, are in line with earlier reports.^33,48^ In the newly constructed double mutant, ELL/AMW, the opposing effects of the two mutations largely canceled one another. This is particularly evident at the 565 and 665/635 nm wavelengths, where the charge recombination kinetics for the ELL/AMW and WT RCs are almost identical (Figure 4 A&C).

Finally, it should be noted that in order to obtain a fully satisfactory fit for the ELL RC, an additional kinetic component of 75 ps had to be added (Figure 2C). Its amplitude was very small and the shape of its DADS resembled the shape of the 160 ps DADS for the AMW RC (compared in Figures S1 and S2). This indicates again a small fraction of the slow ET from B_A_^–^ to H_A_^–^. It is difficult to judge if the minor 75/160 ps processes are natural or artificially induced by isolation/storage procedures.

### Target Analysis—Species-Associated Difference Spectra

**Figure 5:**
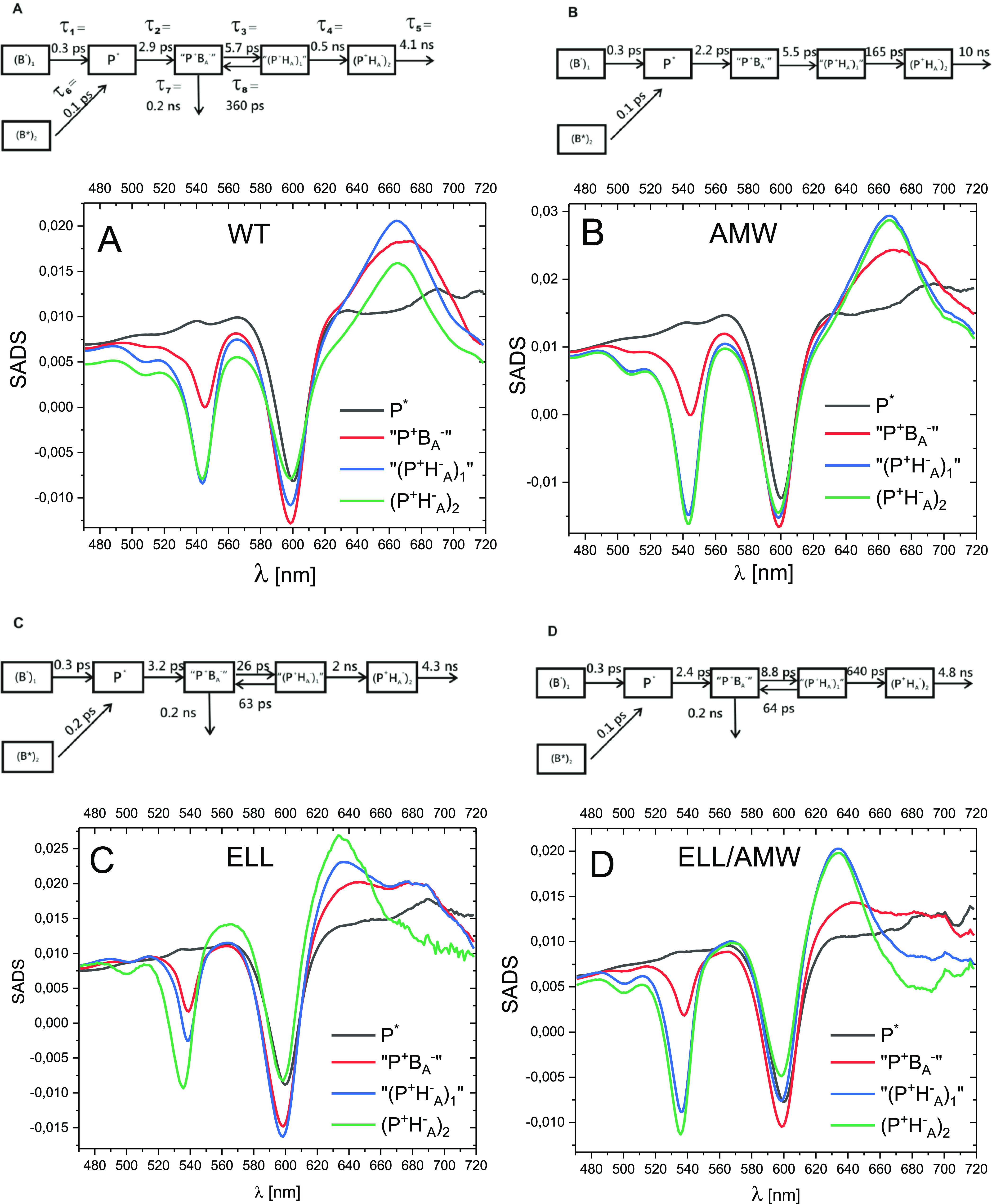
SADS of the WT and three mutant RCs.

The results of global analysis (Figure 2) indicated, in all the samples, a biphasic charge separation with lifetimes of ∼2.5 and ∼6 ps associated mostly with the formation of P^+^B_A_^–^ and P^+^H_A_^–^ states, respectively. This encouraged us to propose a physical model with a separate compartment depicting a pure P^+^B_A_^–^ state. A compartmental model consistent with the global analysis and yielding interpretable spectra, applied to all four RCs, is shown in Figure 5. Two compartments depicting the excited state of B, B_1_^*^ (80% of initial population) and B_2_^*^ (20% of initial population), were necessary to reconstruct the two sub-picosecond phases resolved in the global analysis. Four more compartments were introduced: P* (excited state of the primary donor), “P^+^B_A_^–^” (a dominant P^+^B_A_^–^ state), and two sequentially formed P^+^H_A_^–^ states: “(P^+^H_A_^–^)_1_” and (P^+^H_A_^–^)_2_. Different forms of the P^+^H_A_^–^ state relating to different relaxation states of the protein environment have been proposed previously.^13,14,21,22,31,43,64,65^

**Figure 6:**
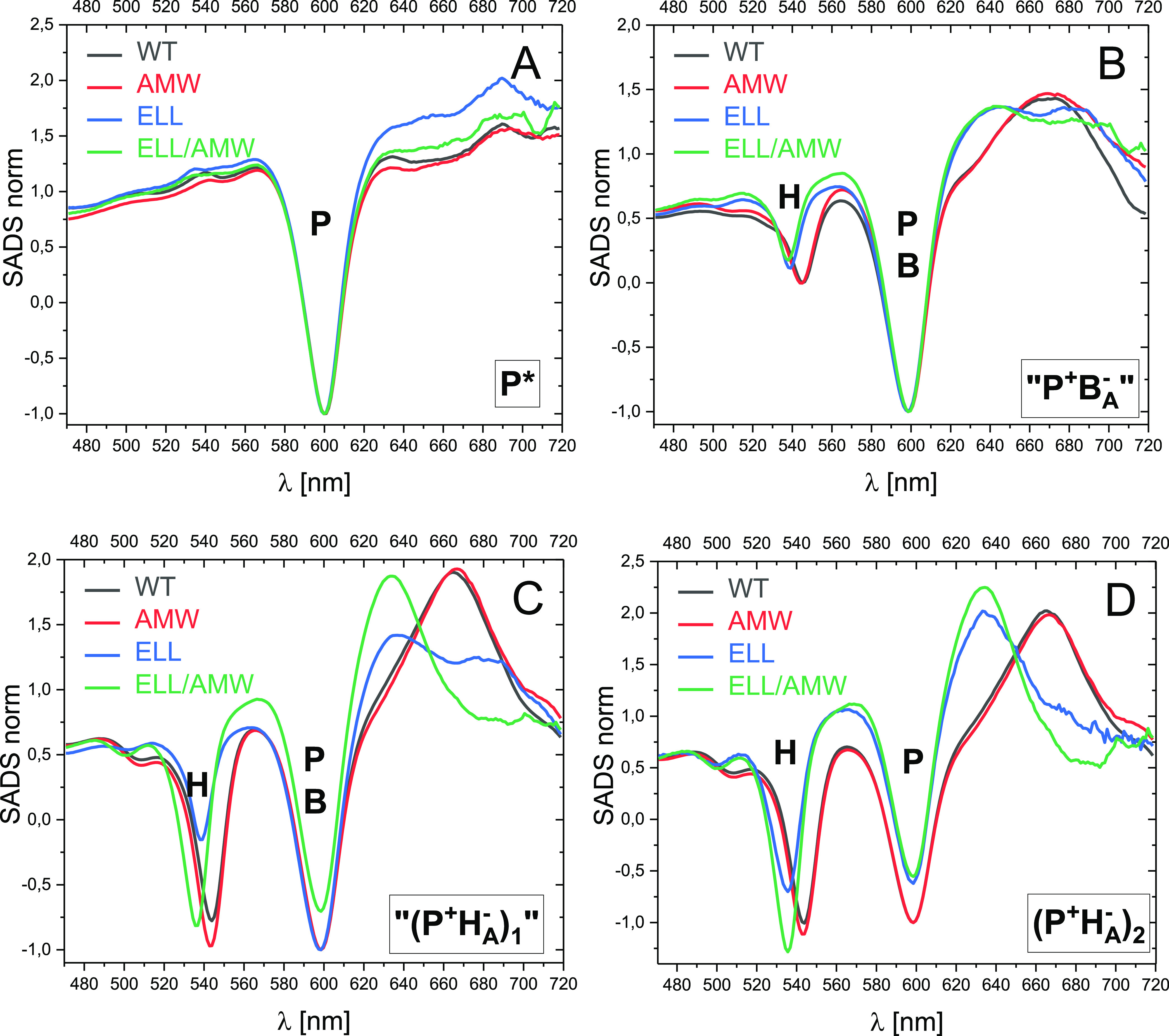
Comparisons of the line shapes of analogous SADS for the four RCs. (A,B) spectra of the compartments P* and “P^+^B_A_^–^”, respectively, normalized at ∼600 nm and (C,D) spectra of the compartments “(P^+^H_A_^–^)_1_” and (P^+^H_A_^–^)_2_ normalized in such a way that the depth of the ∼600 nm PB band relative to the amplitude at 565 nm is the same for all the samples.

The results of the target analysis is presented in a form of SADS (Figure 6). Unlike DADS which are the spectra of individual kinetic components, SADS are the difference spectra of individual transient species relative to the ground-state absorption spectrum of the samples: thus PB of the ground-state absorption is represented by negative bands, whereas transient absorption is represented by positive bands. Overall, the shapes of the respective SADS for all the four RCs were rather similar to one another (Figure 6). The shape of the P* spectrum was almost identical for the four RCs and showed PB exclusively at 600 nm, as expected (Figure 6A). Additionally, one can see a very strong ESA “baseline” that was tilted up toward longer wavelengths and covered the whole spectral region investigated.

The “P^+^B_A_^–^” SADS were also similar for all the RCs, and particularly so within the pairs WT&AMW RC and ELL&ELL/AMW RC (Figure 6B). Interestingly, however, they show PB not only at ∼600 nm, as was initially expected for the pure P^+^B_A_^–^ state, but also additionally an H_A_ PB band at 535 nm (ELL&ELL/AMW RC) or at 544 nm (WT&AMW RC). It was notable that the shapes of these SADS for the WT, ELL, and ELL/AMW RCs resembled the shapes of the 490/590 ps DADS (Figure 3C). Moreover, for the AMW RC, the SADS was similar to that resolved for the WT RC, thus making us confident that the proposed compartmental model is proper. Similarly, as was noted above, when describing the 490/590 ps DADS, the PB signal at ∼600 nm was about 3 times larger than the PB signal at 535/544 nm. This observation, together with the assumption that the differential extinction coefficient for H_A_^–^/H_A_ at ∼540 nm is the same as those for B_A_^–^/B_A_ and P^+^/P at ∼600 nm, indicates that the “P^+^B_A_^–^” SADS is equally contributed to by the states P^+^B_A_^–^ and P^+^H_A_^–^ in all the samples under study (see above). This equal contribution again indicates the isoenergetic character of these two states. In such a case, the latter state could be consequently labeled (P^+^H_A_^–^)_0_ in order to distinguish it from the states “(P^+^H_A_^–^)_1_” and (P^+^H_A_^–^)_2_ resolved in our model (Figure 5). Thus, the compartment “P^+^B_A_^–^” would be an equilibrated state P^+^B_A_^–^ ↔ (P^+^H_A_^–^)_0_. Because we were not able to separately resolve the states P^+^B_A_^–^ and (P^+^H_A_^–^)_0_, we think that such an equilibrium is realized by very fast forward and backward ETs between B_A_ and H_A_. In the case of the extremely fast ET between these two species, the electron may be regarded as delocalized over B_A_ and H_A_. In such a case, the state “P^+^B_A_^–^” should be better labeled as P^+^(B_A_H_A_)_0_^–^. An alternative explanation for the mixed character of the state “P^+^B_A_^–^” could be the faster formation of the state P^+^H_A_^–^ in a fraction of RCs showing an alternative, faster, charge separation pathway: B* → B^+^H_A_^–^ → P^+^H_A_^–^.^61,62^ However, because this pathway was observed only for open WT RCs at 77 K and in a specific mutant, we regard this explanation as less likely. Additionally, the (quasi)isoenergetic equilibrium P^+^B_A_^–^ ↔ P^+^H_A_^–^ lives for up to ∼500 ps (Figure 3C) that would be difficult to reconcile with the heterogeneous origin of the state “P^+^B_A_^–^”.

The third SADS labeled “(P^+^H_A_^–^)_1_” most likely also does not represent a pure state for any of the RCs. This can be judged from the comparison of the shapes of these SADS (Figure 6C) with those of “P^+^B_A_^–^” (Figure 6B) and (P^+^H^–^)_2_ (Figure 6D) for the respective RC. The amount of H_A_^–^ contribution revealed by the amplitude of the 540 nm band relative to that of the ∼600 nm band systematically increased from Figure 6B through 6C to 6D for each of the samples. The (P^+^H^–^)_2_ SADS is believed to be a relatively pure relaxed form of P^+^H_A_^–^ (Figure 6D). For the WT and AMW samples, the (P^+^H_A_^–^)_2_ SADS were highly similar to one another, and both showed PB at ∼540 nm (assigned to H_A_^–^) that was slightly deeper than that at ∼600 nm (assigned to P^+^; Figure 6D). In the previous reports, similar spectral features were interpreted as indicating a pure relaxed state P^+^H_A_^–^.^32,48^ Although the (P^+^H^–^)_2_ SADS of the ELL RC is somewhat different due to the specific mutation affecting H_A_, the relationship between the minima of the ∼540 and ∼600 nm bands was similar to those in the WT and AMW RCs (Figure 6D). The (P^+^H^–^)_2_ SADS for the ELL/AMW RC is characterized by a somewhat deeper band at ∼540 nm but this may be a synergistic effect of the double mutation.

Returning to the “(P^+^H_A_^–^)_1_” SADS (Figure 6C), its shape was intermediate between those of the “P^+^B_A_^–^” and (P^+^H_A_^–^)_2_ SADS (Figure 6B,D). This is most obvious for the ELL RC (compare the amplitudes of the three spectra at ∼540 nm or at ∼635 nm in Figure 5C) but was also seen for the remaining RCs. These intermediate shapes of the “(P^+^H_A_^–^)_1_” spectra indicate the admixture of the state P^+^B_A_^–^ in the “(P^+^H_A_^–^)_1_” compartment.

Regarding the mixed character of the states “P^+^B_A_^–^” and “(P^+^H_A_^–^)_1_”, it should be noted that a similar mixing was proposed previously for open *R. sphaeroides* RCs by Zhu et al.^66^ and explained in the context of adiabatic ET from P* to (B_A_H_A_). We cannot completely rule out the possibility that even more complex modeling, including RC heterogeneity, could result in the resolution of exclusively pure P^+^B_A_^–^ and P^+^H_A_^–^ states, as was obtained in an alternative modeling of the simpler system of open RCs with a neglected charge recombination.^66^ However, closed RCs, particularly those clearly showing P^+^B_A_^–^ → PB_A_ charge recombination on a time scale of hundreds of picoseconds, may favor the delocalization of an electron between B_A_^–^ and H_A_^–^ much more than open RCs do.

### Target Analysis—Intrinsic Lifetimes

Because the common compartmental model generally yielded similar shapes of the respective SADS for the different RCs (Figure 6), significant differences in their kinetics (Figure 4) have to be accounted for by the differences in the intrinsic rate constants or their reciprocals, intrinsic lifetimes, connecting the individual compartments (see the compartmental models in Figure 5). The sub-picosecond lifetimes of ∼100–300 fs (τ_1_ and τ_6_) accounted for B* → P* energy transfer in all the samples. The lifetime of the P* → “P^+^B_A_^–^” charge separation (τ_2_) ranged from ∼2 to ∼3 ps. The next forward reaction “P^+^B_A_” → “(P^+^H_A_^–^)_1_” was accompanied by a back reaction in all the RCs except AMW (Figure 5B). The lack of this back reaction in the AMW RC may be explained by the lack of the electrostatic repulsion between the missing Q_A_^–^ and H_A_^–^ (such a repulsion is present ELL and WT RCs). The lifetime of the “P^+^B_A_^–^” → “(P^+^H_A_^–^)_1_” reaction (τ_3_) in the WT and AMW RC was 5.5–5.7 ps and the lifetime of the back reaction in the WT RC was 360 ps (τ_8_). The ratio of these lifetimes for the WT RC yields a significant free energy gap, Δ*G*, of 104 mV between these states (Table 1) estimated from the formula
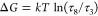
3

**Table 1:** Comparison of the Parameters of the Equilibria “P^+^B_A_^–^” ↔ “(P^+^H_A_^–^)_1_”^a^ and P^+^B_A_^–^ ↔ P^+^H_A_^–^ [within the Compartment “(P^+^H_A_^–^)_1_”] for the Samples under Study

RC	[“(P^+^H_A_^–^)_1_”]/[“P^+^B_A_^–^”] = τ_8_/τ_3_^b^	[“P^+^B_A_^–^”]_rel_^c^ [%]	[“P^+^H_A_^–^”]_rel_^c^ [%]	Δ*G*^d^ [mV]	[P^+^B_A_^–^]_rel_^e^ [%]	[P^+^H_A_^–^]_rel_^e^ [%]	Δ*G*_1_^f^ [mV]
WT	63	1.6	98.4	104	2.8	97.8	88
AMW					5	95	74
ELL	2.4	29	71	22	30	70	21
ELL/AMW	7.3	12	88	50	14	86	45

a “P^+^B_A_^–^” and “(P^+^H_A_^–^)_1_” denote the initial and secondary charge-separated states, respectively, both composed of unresolved virtual pure states P^+^B_A_^–^ and P^+^H_A_^–^.

b Parameters [“P^+^B_A_^–^”] and [“(P^+^H_A_^–^)_1_”] are the equilibrium concentrations of the respective states included in the target analysis in Figure 5; τ_3_ and τ_8_ are lifetimes defined in scheme A in Figure 5.

c Parameters [“P^+^B_A_^–^”]_rel_ and [“P^+^H_A_^–^”]_rel_ are the relative populations of the states “P^+^B_A_^–^” and “(P^+^H_A_^–^)_1_” that are in equilibrium with one another.

d Δ*G* is the absolute value of the free energy gap between the states “(P^+^H_A_^–^)_1_” and “P^+^B_A_^–^”, estimated from eq 3.

e Parameters [P^+^B_A_^–^]_rel_ and [P^+^H_A_^–^]_rel_ are the relative populations of the states P^+^B_A_^–^ and P^+^H_A_^–^ that are in equilibrium with one another within the state “(P^+^H_A_^–^)_1_” [or P^+^(B_A_H_A_)_1_^–^].

f Δ*G*_1_ is the absolute value of the free energy gap between the states P^+^B_A_^–^ and P^+^H_A_^–^ that contribute to the state “(P^+^H_A_^–^)_1_”, as estimated from eq S5.

This gap results in a population of only 1.6% of the more energetic state “P^+^B_A_^–^”. Still, the back reaction is necessary, together with “P^+^B_A_^–^” → PB_A_ charge recombination characterized by lifetime τ_7_ = 200 ps, in order to justify the “leakage” of the “P^+^B_A_^–^” state clearly demonstrated by the 490 ps DADS (Figures 2A and 3C). This value of 200 ps was taken from the literature^32,67,68^ and was fixed for all the samples in our modeling. The value of 490 ps is limited by the 500 ps lifetime (τ_4_), characterizing the next forward reaction, “(P^+^H_A_^–^)_1_” → (P^+^H_A_^–^)_2_. Overall, four intrinsic lifetimes (τ_3_, τ_4_, τ_7_, and τ_8_) define together the efficiency of the “P^+^B_A_^–^” → PB_A_ charge recombination and an apparent lifetime of the “P^+^B_A_^–^” state (Figures 5A and 3C). The second route of charge recombination, (P^+^H_A_^–^)_2_ → PH_A_, without the involvement of the state “P^+^B_A_^–^”, was characterized by the lifetime τ_5_ = 4.1 ns (Figure 5A).

In the AMW RC, the free energy gap between the states “P^+^B_A_” and “(P^+^H_A_^–^)_1_” is expected to be significantly larger than that for the WT RC due to the lack of an electrostatic repulsion between Q_A_^–^ and H_A_^–^ in the former sample. Because of this, and because of the very fast “P^+^B_A_” → “(P^+^H_A_^–^)_1_” reaction (5.5 ps), the “P^+^B_A_^–^” → PB_A_ charge recombination characterized by a lifetime τ_7_ of 200 ps could have been neglected in the model (Figure 5B) without the impairment of the fit quality. The “(P^+^H_A_^–^)_1_” → (P^+^H_A_^–^)_2_ reaction with a 165 ps intrinsic lifetime is related with the small 160 ps DADS shown in Figure 2B and with a very small spectral evolution shown in Figure 5B. In the AMW RCs, the only effective route of charge recombination is (P^+^H^–^)_2_ → PH which takes 10 ns (Figure 5B). The lack of the fast recombination route via the state P^+^B_A_^–^, together with the slowest direct (P^+^H_A_^–^)_2_ → PH_A_ recombination, is the reason why AMW is the mutant with the slowest charge recombination kinetics (Figure 4).

Conversely, charge recombination is the fastest in the ELL RC (Figure 4). This is reflected by the specific combination of τ_3_, τ_4_, and τ_8_ lifetimes. First, the ratio of forward and backward lifetimes, τ_8_/τ_3_ = 2.4, is much smaller than that for the WT (63; Table 1). Thus, the population of the state “P^+^B_A_^–^”, being in equilibrium with “(P^+^H_A_^–^)_1_”, is much larger (29%) than in the WT RC (1.6%; Table 1). Moreover, the “(P^+^H_A_^–^)_1_” → (P^+^H_A_^–^)_2_ reaction lifetime (τ_4_) is 2 ns, 4 times larger than that for the WT RC. These two factors ensure together that the equilibrated state “P^+^B_A_” ↔ “(P^+^H_A_^–^)_1_” decays very effectively via “P^+^B_A_^–^” → PB_A_ charge recombination. Consequently, the competitive alternative charge recombination route, (P^+^H^–^)_2_ → PH, is much less effective, in line with much lower amplitudes of the 4.5 ns DADS compared to the 590 ps DADS (Figure 2C). As a result, the overall charge recombination kinetics in the ELL RC are dominated by the 590 ps component (Figures 2C and 4). At this point, it should be noted that the common compartmental model applied to the ELL sample (Figure 5) results in six DADS (see Figure S3 and the corresponding section in the Supporting Information) and not seven as described above in the Global Analysis section. The seven-exponential global analysis model gave a somewhat better fit due to the introduction of the small 75 ps component (Figure 2C). However, we were not able to generate reliably a more complex compartmental model, with an additional compartment, reproducing the seven lifetimes obtained in the global analysis. Consequently, the relatively large value of τ_3_ = 26 ps (Figure 5C) may be an overestimation resulting from the 6-compartmental model, whereas the seven-exponential global fit suggests a much faster second phase of charge separation, of the order of 6 ps (Figure 2C), similar to those for the remaining RCs (Figure 2).

For the ELL/AMW RC (Figure 5D), the value of τ_8_/τ_3_ is 7.3, which is 3 times larger than the 2.4 obtained for the ELL RC (Table 1), and therefore the “P^+^B_A_” ↔ “(P^+^H_A_^–^)_1_” equilibrium is shifted more toward “(P^+^H_A_^–^)_1_” in the double mutant. On the other hand, τ_4_ = 640 ps which is 3 times less than the 2 ns seen for the ELL RC. Both these differences make the “fast route” of charge recombination, via the state “P^+^B_A_^–^”, less efficient in the ELL/AMW RC than in the ELL RC. Consequently, charge recombination for the ELL/AMW RC occurs mostly via the “slow route” [direct (P^+^H_A_^–^)_2_ → PH_A_ recombination; see also the amplitudes of the 510 ps and 4.8 ns DADS in Figure 2D] and thus is slower than that for the ELL RC (Figure 4). When comparing the lifetimes obtained for the ELL/AMW RC with those for the WT, one can see that the values of the τ_8_/τ_3_ fraction (Table 1) and τ_4_ promote a higher efficiency of charge recombination via the “slow route” in the WT RC [(P^+^H_A_^–^)_2_ → PH_A_]. On the other hand, the direct (P^+^H_A_^–^)_2_ → PH_A_ charge recombination lifetime is somewhat slower for the ELL/AMW RC (τ_5_ = 4.8 ns) than that for the WT (4.1 ns). The net result of these differences is generally faster charge recombination dynamics in the ELL/AMW RC than in the WT (compare DADS in Figure 2A,D and kinetics in Figure 4B), but at some wavelengths, the kinetics for both samples are very similar to one another (Figure 4A,C). A clear effect of the double mutation, compared to the AMW RC, is an acceleration of the direct charge recombination, (P^+^H_A_^–^)_2_ → PH_A_ (τ_6_), from 10 ns (characteristic of AMW) to 4.8 ns.

### Energetic Model

The opposing effects of the ELL and AMW mutations on the kinetics of P^+^H_A_^–^ → PH_A_ recombination largely cancel one another in the double mutant ELL/AMW. This observation supports the model in which the overall P^+^H_A_^–^ → PH_A_ recombination dynamics are controlled by the free energy gap between the states P^+^H_A_^–^ and P^+^B_A_^–^, independent of the nature of modifications leading to the change in this free energy gap. In the AMW RC, the free energy gap is increased relative to that in the WT RC (Figure 1A) by removing the repulsive electrostatic interaction between Q_A_^–^ and H_A_^–^ that is characteristic for a WT RC with a permanently reduced quinone, Q_A_^–^. This was achieved by the removal of the quinone Q_A_ from its binding pocket through the AMW mutation. On the other hand, in the ELL RC, the free energy gap is decreased relative to that in the WT RC by removing the hydrogen bond between a glutamate side chain and H_A_, which in the WT RC stabilizes the state P^+^H_A_^–^.^33^ Both mutations affect H_A_ on its distal side from P and B_A_, and they should therefore not affect the properties of B_A_ and P (Figure 1A). Indeed, whereas the ELL mutation clearly shifts the H_A_ Q_x_ and anionic absorption bands (Figure 3B–D), no evident effect of this or the AMW mutation on the B_A_ or P bands was observed. Thus, we propose that the cancelation of the opposing effects of the ELL and AMW mutations on the charge recombination kinetics in the double mutant originates from the cancelation of their influence on the free energy level of the state P^+^H_A_^–^ relative to that of P^+^B_A_^–^ (Figure 1C). However, it was shown previously that the state P^+^H_A_^–^ evolves in time due to the relaxation of the protein environment, leading to a gradual free energy gap increase between the states P^+^H_A_^–^ and P^+^B_A_^–^.^13,14,21,22,31,43,64,65^ Therefore, the question arises of how to relate the temporal evolution of this gap with the influence of the mutations on it.

**Figure 7:**
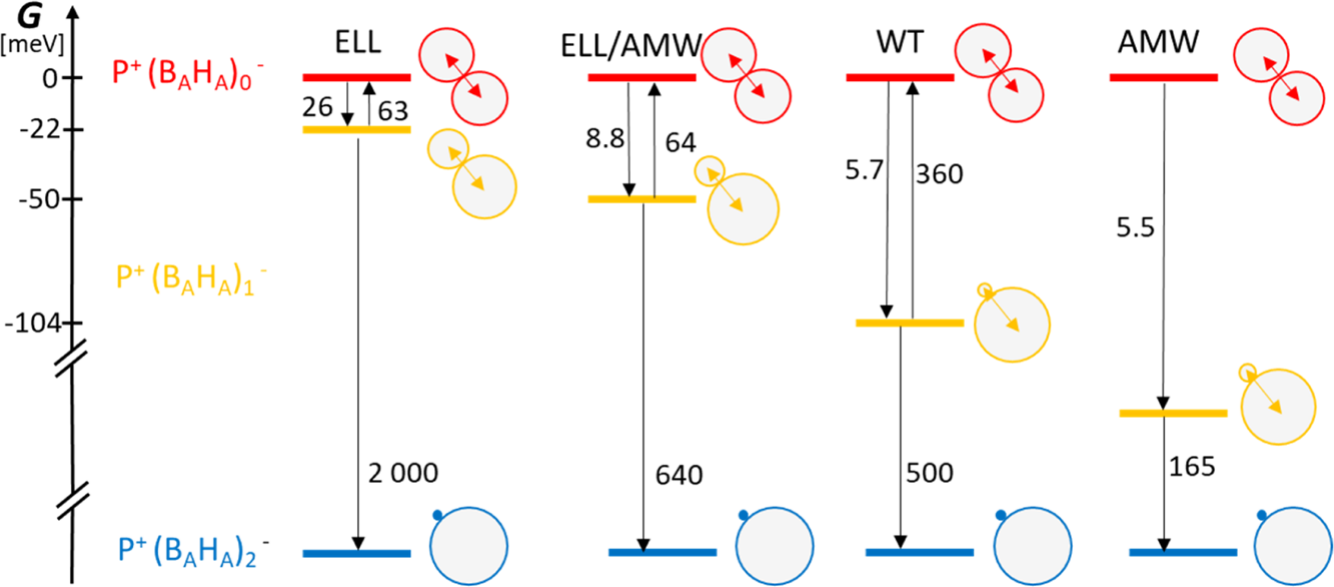
Energetic model of relaxation of the charge-separated state P^+^(B_A_H_A_)^−^ for the four RCs. The initially formed charge-separated state is in red, the intermediate state is in yellow, and the finally resolved charge-separated state is in blue. The circles represent B_A_ (upper left circle in each pair) and H_A_ (lower right circle in each pair), and their areas are proportional to the probability of the localization of the electron on B_A_ or H_A_. The numerical values for these probabilities are given in Table 1 (columns [P^+^B_A_^–^]_rel_ and [P^+^H_A_^–^]_rel_). The numbers in black at the arrows are the molecular lifetimes (in ps) obtained from the target analysis (see Figure 5). Note that the free energy level of the state P^+^(B_A_H_A_)_0_^–^ was arbitrarily set at 0 meV, while the free energy level of the state P^+^(B_A_H_A_)_2_^–^ (and P^+^(B_A_H_A_)_1_^–^ for AMW) was not determined.

In our target analysis, we were not able to resolve a pure P^+^B_A_^–^ state. In all samples, the compartment “P^+^B_A_^–^” contains a contribution from the state P^+^H_A_^–^ (Figure 5), as indicated by a negative band at ∼540 nm due to H_A_ PB. Similarly, the compartment “(P^+^H_A_^–^)_1_” describes not a pure P^+^H_A_^–^ state but one which contains a contribution from P^+^B_A_^–^, as noted above. Therefore, in our energetic model (Figure 7), consistent with the target analysis, we replaced the labels “P^+^B_A_^–^” and “(P^+^H_A_^–^)_1_” with the labels P^+^(B_A_H_A_)_0_^–^ and P^+^(B_A_H_A_)_1_^–^, respectively. For consistency, we also replaced the label (P^+^H_A_^–^)_2_ with P^+^(B_A_H_A_)_2_^–^. These replacements (except for the last one) are not only formal ones but they bear interpretational consequences. In such an approach, each of the discrete states P^+^(B_A_H_A_)_*i*_^–^ represents a charge-separated state related to a different protein relaxation state. In each of these states, the electron is delocalized over B_A_ and H_A_. However, the degree of this delocalization evolves in time after the excitation (Figure 7). In the state P^+^(B_A_H_A_)_0_^–^, where the contributions of the states P^+^B_A_^–^ and P^+^H_A_^–^ were shown above to be roughly the same for all the RCs (Figure 6B), the delocalization ratio is ∼50:50. This delocalization is shifted toward H_A_^–^ in the state P^+^(B_A_H_A_)_1_^–^, and the electron is assumed to be fully localized on H_A_ in the state P^+^(B_A_H_A_)_2_^–^. In order to estimate the delocalization ratio between B_A_ and H_A_ in the state P^+^(B_A_H_A_)_1_^–^, we compared the amplitudes of the “(P^+^H_A_^–^)_1_” SADS at ∼540 and ∼600 nm with those of the corresponding (P^+^H_A_^–^)_2_ SADS (see Figure S4 and the corresponding section in the Supporting Information for details). As a result, the B_A_/H_A_ delocalization ratio was estimated to be ∼3:97 for the WT RC, 5:95 for the AMW RC, 30:70 for the ELL RC, and 14:86 for the ELL/AMW RC (Table 1). In Figure 7, the electron localization on B_A_ versus H_A_ is proportional to the areas of circles representing the two molecules. We identify the electron delocalization ratios with the population ratios of the virtual pure states P^+^B_A_^–^ and P^+^H_A_^–^ within the state P^+^(B_A_H_A_)_1_^–^, and thus the delocalization ratios allow the estimation of the free energy gaps, Δ*G*_1_ (eq S5), between these virtual states (Table 1). On the other hand, Figure 7 shows the free energy gaps between the states P^+^(B_A_H_A_)_0_^–^ and P^+^(B_A_H_A_)_1_^–^ obtained directly from the target analysis (Table 1, eq 3).

It is not clear whether the mixing between the states P^+^B_A_^–^ and P^+^H_A_^–^ within the states P^+^(B_A_H_A_)_0_^–^ and P^+^(B_A_H_A_)_1_^–^ is strictly quantum mechanical mixing or whether it comes from fast transitions between pure P^+^B_A_^–^ and P^+^H_A_^–^ states. We tend to think that initially, in the state P^+^(B_A_H_A_)_0_^–^, the electron is delocalized quantum mechanically between B_A_^–^ and H_A_^–^ in line with the suggestion by Zhu et al.^66^ The state P^+^(B_A_H_A_)_1_ may depict the situation of fast transitions between pure P^+^B_A_^–^ and P^+^H_A_^–^ states.

The model shown in Figure 7 indicates that the initial state P^+^(B_A_H_A_)_0_^–^, in which the virtual pure states P^+^B_A_^–^ and P^+^H_A_^–^ are isoenergetic, appears in all the RCs under study. Apparently, the mutations do not affect the initial energy level of P^+^H_A_^–^ relative to that of P^+^B_A_^–^. This may seem surprising. However, it should be noted that charge separation is not heavily affected by the mutations (Figure 3A), and it may be that equal charge distribution between B_A_^–^ and H_A_^–^ is a requirement for the fast charge separation observed both in the WT RC and in the mutant complexes. Investigation by other techniques would be valuable to verify the isoenergetic character of initial P^+^B_A_^–^ and P^+^H_A_^–^ states. Measurements of delayed fluorescence could provide information on the free energies of all the states relative to P*. The energies might be rationalized by electrostatic calculations and molecular dynamics (MD) simulations. MD simulations also could help to elucidate the relaxations of the protein (see below).

Temporal evolution from the state P^+^(B_A_H_A_)_0_^–^ to the state P^+^(B_A_H_A_)_1_^–^ and then to P^+^(B_A_H_A_)_2_^–^ is an individual feature of each of the RCs (Figure 7). The relaxation process is approximated by two exponential phases: a fast one of 5.5–26 ps lifetime and a slow one of 165–2000 ps lifetime. The fast phase of relaxation for the WT, AMW, and ELL/AMW RCs, of 5.5–8.8 ps lifetime, occurs in parallel with the (partial) localization of the electron on H_A_, proposed to be a consequence of very rapid protein relaxation. Consequently, we think that this phase of a few picoseconds, classically described as a second phase of charge separation, is in fact the protein response to a single step charge separation reaction P* → P^+^(B_A_H_A_)_0_^–^ occurring with a ∼2–3 ps lifetime (Figures 2 and 5). The model shows that this phase is slower for ELL (26 ps). This could be an effect of particularly effective electrostatic repulsion between Q_A_^–^ and the electron to be transferred from P* to the pair B_A_H_A_ in this mutant. However, it is also possible that the large value of 26 ps results from the inaccuracy of the target model containing only six compartments, whereas in the seven-component global fit, the 5.7 ps phase could have been resolved similar to those in the remaining samples (Figure 2C).

Unlike the unrelaxed state P^+^(B_A_H_A_)_0_^–^, the properties of the first relaxed state P^+^(B_A_H_A_)_1_^–^ is clearly mutation-dependent. Its free energy level is clearly shifted up in the ELL RC and shifted down in the AMW RC, relative to its position in the WT RC (Figure 7). In the case of the ELL/AMW RC, this level is in between those of the ELL and AMW RCs in line with predictions summarized in Figure 1C. The estimation of the free energy gaps between the states P^+^(B_A_H_A_)_0_^–^ and P^+^(B_A_H_A_)_1_^–^, Δ*G*, was possible due to the back reactions modeled in the target analysis (Figures 5 and 7). We postulate that this reversibility holds for protein relaxation states and not for the B_A_^–^ ↔ H_A_^–^ equilibrated ET. Different degrees of localization of the electron on H_A_ in these two states are only a consequence of two different protein relaxations or conformational states. The observed correlation between the Δ*G* gap and the degree of electron localization on H_A_ (the larger Δ*G* is the stronger is the localization of the electron on H_A_) is a secondary effect of the protein relaxation.

In our model, the states P^+^(B_A_H_A_)_0_^–^ and P^+^(B_A_H_A_)_1_^–^ show the possibility of charge recombination by the back ET from B_A_^–^ to the ground orbital of P^+^, and this reaction is particularly effective when the Δ*G* gap is small. Thus, the Δ*G* gap, together with the second relaxation step lifetime (165–2000 ps; Figure 7), controls the branching between the fast charge recombination route (via P^+^B_A_^–^) and the slow one (direct P^+^H_A_^–^ → PH_A_ charge recombination). The Δ*G* gap is particularly large (and unmeasurable because of the unresolved back reaction) in the AMW RC. Consequently, the only charge recombination in this sample is the direct one and thus is particularly slow.

In summary, we were able to resolve two phases of the protein relaxation—a faster one (5.5–26 ps) and a slower one (165–2000 ps). The second phase of relaxation leads to the state P^+^(B_A_H_A_)_2_^–^ that was assumed to recombine only directly within ∼4–5 ns (WT, ELL, and ELL/AMW RCs) or ∼10 ns (AMW RC). However, due to the time window of the transient absorption experiment being limited to ∼3 ns, the values of lifetimes of 4–10 ns may be rather inaccurate. Indeed, in addition to the sub-nanosecond components, two more recombination phases have been resolved, ranging from ∼1 to ∼20 ns, in experiments performed in wider time windows.^21,22,33,69^ Thus, it is very likely that the single ∼4–10 ns phases reported in this paper are actually an approximation of two different phases occurring over a nanosecond time scale. It was proposed before that the relaxed P^+^H_A_^–^ state decaying on a few nanosecond time scale is kinetically faster than the next P^+^H_A_^–^ state (decaying within ≳10 ns) due to equilibrium with the state P^+^B_A_^–^ persisting up to the nanosecond time scale.^69^

### Primary Charge Separation

It is interesting to compare the primary charge separation in our samples with that reported for open WT RCs, that is, with a neutral Q_A_. There has been a long-standing discussion on the nature of this reaction. It is commonly accepted that this reaction is biphasic with the primary step leading to the ET from P* to B_A_ and then from B_A_^–^ to H_A_ (see eq 1). According to this equation, B_A_^–^ decays faster than it is formed and this is the reason why it is so difficult to detect it in open WT RCs. However, in closed RCs, it is possible to form the state B_A_^–^ with a higher efficiency (Figure 2). This is further confirmed by the target modeling showing that unlike in open RCs, the depopulation of the state “P^+^B_A_^–^” (τ_3_) is slower than its population (τ_2_) for all the samples. For all the samples but the AMW RC, this effect could be related to an electrostatic interaction between electrons on Q_A_^–^ and on (B_A_H_A_)^−^. However, the existence of this effect for the AMW RC also makes the previous assignment uncertain. Therefore, for the WT and AMW RCs, we also tried to exchange the τ_3_ and τ_2_ values in our target analysis, while leaving all the remaining intrinsic lifetimes relatively unchanged. This was done in order to make sure that τ_3_ is indeed larger than τ_2_. Such an exchange resulted in DADS (Figure S5C,D) of equally good quality and similar apparent lifetimes as those obtained for original fits (Figure 2A,B). However, the resulting amplitudes of the “P^+^B_A_^–^” SADS were meaningless (Figure S5A,B).

## Conclusions

We have shown that a combination of two single point mutations, one of which accelerates charge recombination and the other decelerates it, leads to significant cancelation of the single mutation effects. Additionally, we propose a new view of both the primary charge separation and the charge recombination in closed bacterial photosynthetic RCs. They may be summarized in the following way
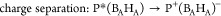

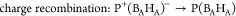


In this model, following charge separation, a 50:50 B_A_/H_A_ electron delocalization ratio gradually shifts toward a higher localization of the electron on H_A_ due to protein dynamics. This shift is accompanied by a slowing of charge recombination. The question remains regarding what is the cause and what is the effect: whether the charge-separated state induces a passive dielectric response of the protein^69^ or the protein dynamics, triggered by excitation, forces the delocalization shift.^42^ A mixture of both these mechanisms is also possible. We conclude that irrespectively of the exact mechanism the protein dynamics affects the energetics and kinetics of the primary charge separation and charge recombination.

## Supporting Information Available

Comparison of the 5.5-ps and 160-ps DADS for the AMW RC; 75-ps DADS of the ELL RC; comparison of six- and seven-exponential global fits for the ELL sample; estimation of the relative contribution of the states P^+^B_A_^–^ and P^+^H_A_^–^ within the compartment “(P^+^H_A_^–^)_1_”; results of target and global analysis for the WT and AMW RCs with alternative values of τ_3_ and τ_4_ (PDF)

## References

[ref1] Anoxygenic Photosynthetic Bacteria; Blankenship R. E., Madigan M. T., Bauer C. E., Eds.; Kluwer Academic Publishers: Dordrecht/Boston/London, 1995.

[ref2] The Purple Phototrophic Bacteria; Hunter C. N., Daldal F., Thurnauer M. C., Beatty J. T., Eds.; Springer: Dordrecht, 2009.

[ref3] Photosystem II: The Light-Driven Water: Plastoquinone Oxidoreductase; Wydrzynski T. J., Satoh K., Eds.; Springer: Dordrecht, 2005.

[ref4] Photosynthetic Reaction Centers in The Biophysics of Photosynthesis; Golbeck J., van der Est A., Eds.; Springer: New York/Heidelberg/Dordrecht/London, 2014.

[ref5] Martin J. L.; Breton J.; Hoff A. J.; Migus A.; Antonetti A. Femtosecond spectroscopy of electron transfer in the reaction center of the photosynthetic bacterium Rhodopseudomonas sphaeroides R-26: Direct electron transfer from the dimeric bacteriochlorophyll primary donor to the bacteriopheophytin acceptor with a time constant of 2.8 0.2 psec. Proc. Natl. Acad. Sci. U.S.A. 1986, 83, 957–961. 10.1073/pnas.83.4.957. 16593659 PMC322989

[ref6] Parson W. W.; Warshel A. Mechanism of Charge Separation in Purple Bacterial Reaction Centers. In The Purple Phototrophic Bacteria; Hunter C. N., Daldal F., Thurnauer M. C., Beatty J. T., Eds.; Springer: Netherlands, 2009.

[ref7] Savikhin S.; Jankowiak R. Mechanism of Primary Charge Separation in Photosynthetic Reaction Centers. The Biophysics of Photosynthesis. Biophysics for the Life Sciences; Golbeck J., van der Est A., Eds.; Springer: New York, 2014; Vol. 11.

[ref8] Allen J. P.; Feher G.; Yeates T. O.; Komiya H.; Rees D. C. Structure of the reaction center from Rhodobacter sphaeroides R-26: the cofactors. Proc. Natl. Acad. Sci. U.S.A. 1987, 84, 5730–5734. 10.1073/pnas.84.16.5730. 3303032 PMC298936

[ref9] Woodbury N. W. T.; Allen J. P. Electron transfer in purple nonsulfur bacteria. In Anoxygenic Photosynthetic Bacteria; Blankenship R. E., Madigan M. T., Bauer C. E., Eds.; Kluwer Academic Publishers: Dordrecht/Boston/London, 1995; p 527.

[ref10] Marchi M.; Gehlen J. N.; Chandler D.; Newton M. Diabatic surfaces and the pathway for primary electron transfer in a photosynthetic reaction center. J. Am. Chem. Soc. 1993, 115, 4178–4190. 10.1021/ja00063a041.

[ref11] Arlt T.; Schmidt S.; Kaiser W.; Lauterwasser C.; Meyer M.; Scheer H.; Zinth W. The accessory bacteriochlorophyll: a real electron carrier in primary photosynthesis. Proc. Natl. Acad. Sci. U.S.A. 1993, 90, 11757–11761. 10.1073/pnas.90.24.11757. 11607443 PMC48063

[ref12] Zinth W.; Wachtveitl J. The First Picoseconds in Bacterial Photosynthesis?Ultrafast Electron Transfer for the Efficient Conversion of Light Energy. ChemPhysChem 2005, 6, 871–880. 10.1002/cphc.200400458. 15884069

[ref13] Holzwarth A. R.; Müller M. G. Energetics and kinetics of radical pairs in reaction centers from Rhodobacter sphaeroides. A femtosecond transient absorption study. Biochem 1996, 35, 11820–11831. 10.1021/bi9607012. 8794764

[ref14] van Stokkum I. H. M.; Beekman L. M. P.; Jones M. R.; van Brederode M. E.; van Grondelle R. Primary electron transfer kinetics in membrane-bound Rhodobacter sphaeroides reaction centers: a global and target analysis. Biochem 1997, 36, 11360–11368. 10.1021/bi9707943. 9298955

[ref15] Kaufmann K. J.; Dutton P. L.; Netzel T. L.; Leigh J. S.; Rentzepis P. M. Picosecond kinetics of events leading to reaction center bacteriochlorophyll oxidation. Science 1975, 188, 1301–1304. 10.1126/science.188.4195.1301. 17772598

[ref16] Rockley M. G.; Windsor M. W.; Cogdell R. J.; Parson W. W. Picosecond detection of an intermediate in the photochemical reaction of bacterial photosynthesis. Proc. Natl. Acad. Sci. U.S.A. 1975, 72, 2251–2255. 10.1073/pnas.72.6.2251. 1079602 PMC432735

[ref17] Holten D.; Windsor M. W.; Parson W. W.; Thornber J. P. Primary photochemical processes in isolated reaction centers of Rhodopseudomonas viridis. Biochim. Biophys. Acta 1978, 501, 112–126. 10.1016/0005-2728(78)90100-7. 620009

[ref18] Volk M.; Ogrodnik A.; Michel-Beyerle M. E. The Recombination Dynamics of the Radical Pair P^+^H^-^ in External Magnetic and Electric Fields. In Photosynthetic Bacteria; Blankenship R. E., Madigan M. T., BauerAnoxygenic C. E., Eds.; Kluwer Academic Publishers: Dordrecht, Boston, London, The Netherlands, 1995.

[ref19] Shuvalov V. A.; Klimov V. V. The primary photoreactions in the complex cytochrome-P-890 . P-760 (bacteriopheophytin 760) of Chromatium minutissimum at low redox potentials. Biochim. Biophys. Acta 1967, 440, 587–599. 10.1016/0005-2728(76)90044-x. 183814

[ref20] Schenck C. C.; Blankenship R. E.; Parson W. W. Radical-pair decay kinetics, triplet yields and delayed fluorescence from bacterial reaction centers. Biochim. Biophys. Acta 1982, 680, 44–59. 10.1016/0005-2728(82)90315-2.

[ref21] Woodbury N. W. T.; Parson W. W. Nanosecond fluorescence from isolated photosynthetic reaction centers of Rhodopseudomonas sphaeroides. Biochim. Biophys. Acta 1984, 767, 345–361. 10.1016/0005-2728(84)90205-6. 6333897

[ref22] Woodbury N. W.; Parson W. W.; Gunner M. R.; Prince R. C.; Dutton P. L. Radical-pair energetics and decay mechanisms in reaction centers containing anthraquinones, naphthoquinones or benzoquinones in place of ubiquinone. Biochim. Biophys. Acta 1986, 851, 6–22. 10.1016/0005-2728(86)90243-4. 3524681

[ref23] Ogrodnik A.; Keupp W.; Volk M.; Aumeier G.; Michel-Beyerle M. E. Inhomogeneity of Radical Pair Energies in Photosynthetic Reaction Centers Revealed by Differences in Recombination Dynamics of P+HA- When Detected in Delayed Emission and in Absorption. J. Phys. Chem. 1994, 98, 3432–3439. 10.1021/j100064a026.

[ref24] Hartwich G.; Lossau H.; Michel-Beyerle M. E.; Ogrodnik A. Nonexponential Fluorescence Decay in Reaction Centers ofRhodobactersphaeroidesReflecting Dispersive Charge Separation up to 1 ns. J. Phys. Chem. B 1998, 102, 3815–3820. 10.1021/jp973472h.

[ref25] Tang C.-K.; Williams J. C.; Taguchi A. K. W.; Allen J. P.; Woodbury N. W. P+HA- Charge Recombination Reaction Rate Constant in Rhodobacter sphaeroides Reaction Centers is Independent of the P/P+ Midpoint Potential. Biochemistry 1999, 38, 8794–8799. 10.1021/bi990346q. 10393555

[ref26] Shuvalov V. A.; Parson W. W. Energies and kinetics of radical pairs involving bacteriochlorophyll and bacteriopheophytin in bacterial reaction centers. Proc. Natl. Acad. Sci. U.S.A. 1981, 78, 957–961. 10.1073/pnas.78.2.957. 16592980 PMC319924

[ref27] Ridge J. P.; van Brederode M. E.; Goodwin M. G.; van Grondelle R.; Jones M. R. Mutations that modify or exclude binding of the QA ubiquinone and carotenoid in the reaction center from Rhodobacter sphaeroides. Photosynth. Res. 1999, 59, 9–26. 10.1023/a:1006111321083.

[ref28] McAuley K. E.; Fyfe P. K.; Ridge J. P.; Cogdell R. J.; Isaacs N. W.; Jones M. R. Ubiquinone binding, ubiquinone exclusion, and detailed cofactor conformation in a mutant bacterial reaction center. Biochemistry 2000, 39, 15032–15043. 10.1021/bi000557r. 11106481

[ref29] Woodbury N. W.; Becker M.; Middendorf D.; Parson W. W. Picosecond kinetics of the initial photochemical electron-transfer reaction in bacterial photosynthetic reaction centers. Biochemistry 1985, 24, 7516–7521. 10.1021/bi00347a002. 3879185

[ref30] Wang S.; Lin S.; Lin X.; Woodbury N. W.; Allen J. P. Comparative study of reaction centers from purple photosynthetic bacteria: Isolation and optical spectroscopy. Photosynth. Res. 1994, 42, 203–215. 10.1007/bf00018263. 24306562

[ref31] Gibasiewicz K.; Pajzderska M. Primary Radical Pair P+H-Lifetime inRhodobactersphaeroideswith Blocked Electron Transfer to QA. Effect ofo-Phenanthroline. J. Phys. Chem. B 2008, 112, 1858–1865. 10.1021/jp075184j. 18215032

[ref32] Gibasiewicz K.; Pajzderska M.; Ziółek M.; Karolczak J.; Dobek A. Internal Electrostatic Control of the Primary Charge Separation and Recombination in Reaction Centers from Rhodobacter sphaeroides Revealed by Femtosecond Transient Absorption. J. Phys. Chem. B 2009, 113, 11023–11031. 10.1021/jp811234q. 19603803

[ref33] Gibasiewicz K.; Pajzderska M.; Potter J. A.; Fyfe P. K.; Dobek A.; Brettel K.; Jones M. R. Mechanism of Recombination of the P+HA-Radical Pair in MutantRhodobacter sphaeroidesReaction Centers with Modified Free Energy Gaps Between P+BA-and P+HA-. J. Phys. Chem. B 2011, 115, 13037–13050. 10.1021/jp206462g. 21970763

[ref34] Gibasiewicz K.; Pajzderska M.; Dobek A.; Karolczak J.; Burdziński G.; Brettel K.; Jones M. R. Analysis of the Temperature-Dependence of P+HA - Charge Recombination in the Rhodobacter sphaeroides Reaction Center Suggests Nanosecond Temperature-Independent Protein Relaxation. Phys. Chem. Chem. Phys. 2013, 15, 16321–16333. 10.1039/c3cp44187c. 23999896

[ref35] Wang H.; Hao Y.; Jiang Y.; Lin S.; Woodbury N. W. Role of protein dynamics in guiding electron-transfer pathways in reaction centers from Rhodobacter sphaeroides. J. Phys. Chem. B 2012, 116, 711–717. 10.1021/jp211702b. 22148392

[ref36] Frauenfelder H. The Physics of Proteins: An Introduction to Biological Physics and Molecular Biophysics; Springer-Verlag: New York, 2011.

[ref37] Warshel A.; Chu Z.; Parson W. Dispersed polaron simulations of electron transfer in photosynthetic reaction centers. Science 1989, 246, 112–116. 10.1126/science.2675313. 2675313

[ref38] Gehlen J. N.; Marchi M.; Chandler D. Dynamics affecting the primary charge transfer in photosynthesis. Science 1994, 263, 499–502. 10.1126/science.263.5146.499. 17754881

[ref39] Graige M. S.; Feher G.; Okamura M. Y. Conformational gating of the electron transfer reaction QA-*QB -> QAQB-* in bacterial reaction centers of Rhodobacter sphaeroides determined by a driving force assay. Proc. Natl. Acad. Sci. U.S.A. 1998, 95, 11679–11684. 10.1073/pnas.95.20.11679. 9751725 PMC21700

[ref40] McMahon B. H.; Müller J. D.; Wraight C. A.; Nienhaus G. U. Electron transfer and protein dynamics in the photosynthetic reaction center. Biophys. J. 1998, 74, 2567–2587. 10.1016/s0006-3495(98)77964-0. 9591682 PMC1299598

[ref41] Parson W. W.; Warshel A. Dependence of Photosynthetic Electron-Transfer Kinetics on Temperature and Energy in a Density-Matrix Model. J. Phys. Chem. B 2004, 108, 10474–10483. 10.1021/jp0495904.

[ref42] Wang H.; Lin S.; Allen J. P.; Williams J. C.; Blankert S.; Laser C.; Woodbury N. W. Protein dynamics control the kinetics of initial electron transfer in photosynthesis. Science 2007, 316, 747–750. 10.1126/science.1140030. 17478721

[ref43] Pawlowicz N. P.; van Grondelle R.; van Stokkum I. H. M.; Breton J.; Jones M. R.; Groot M. L. Identification of the first steps in charge separation in bacterial photosynthetic reaction centers of Rhodobacter sphaeroides by ultrafast mid-infrared spectroscopy: Electron transfer and protein dynamics. Biophys. J. 2008, 95, 1268–1284. 10.1529/biophysj.108.130880. 18424493 PMC2479572

[ref44] Guo Z.; Woodbury N. W.; Pan J.; Lin S. Protein Dielectric Environment Modulates the Electron-Transfer Pathway in Photosynthetic Reaction Centers. Biophys. J. 2012, 103, 1979–1988. 10.1016/j.bpj.2012.09.027. 23199926 PMC3491703

[ref45] LeBard D. N.; Matyushov D. V. Energetics of Bacterial Photosynthesis. J. Phys. Chem. B 2009, 113, 12424–12437. 10.1021/jp904647m. 19691308

[ref46] LeBard D. N.; Matyushov D. V. Protein-water electrostatics and principles of bioenergetics. Phys. Chem. Chem. Phys. 2010, 12, 15335–15348. 10.1039/c0cp01004a. 20972505

[ref47] LeBard D. N.; Martin D. R.; Lin S.; Woodbury N. W.; Matyushov D. V. Protein dynamics to optimize and control bacterial photosynthesis. Chem. Sci. 2013, 4, 4127–4136. 10.1039/c3sc51327k.

[ref48] Gibasiewicz K.; Białek R.; Pajzderska M.; Karolczak J.; Burdziński G.; Jones M. R.; Brettel K. Weak temperature dependence of P (+) H A (-) recombination in mutant Rhodobacter sphaeroides reaction centers. Photosynth. Res. 2016, 128, 243–258. 10.1007/s11120-016-0239-9. 26942583 PMC4877430

[ref49] McAuley-Hecht K. E.; Fyfe P. K.; Ridge J. P.; Prince S. M.; Hunter C. N.; Isaacs N. W.; Cogdell R. J.; Jones M. R. Structural studies of wild-type and mutant reaction centers from an antenna-deficient strain of Rhodobacter sphaeroides: monitoring the optical properties of the complex from bacterial cell to crystal. Biochemistry 1998, 37, 4740–4750. 10.1021/bi971717a. 9537989

[ref50] Jones M. R.; Fowler G. J. S.; Gibson L. C. D.; Grief G. G.; Olsen J. D.; Crielaard W.; Hunter C. N. Mutants of Rhodobacter sphaeroides lacking one or more pigment-protein complexes and complementation with reaction-centre, LH1, and LH2 genes. Mol. Microbiol. 1992, 6, 1173–1184. 10.1111/j.1365-2958.1992.tb01556.x. 1588816

[ref51] Jones M. R.; Heer-Dawson M.; Mattioli T. A.; Hunter C. N.; Robert B. Site-specific mutagenesis of the reaction centre from Rhodobacter sphaeroides studied by Fourier transform Raman spectroscopy: mutations at tyrosine M210 do not affect the electronic structure of the primary donor. FEBS Lett. 1994, 339, 18–24. 10.1016/0014-5793(94)80376-5. 8313970

[ref52] Bylina E. J.; Kirmaier C.; McDowell L.; Holten D.; Youvan D. C. Influence of an amino-acid residue on the optical properties and electron transfer dynamics of a photosynthetic reaction centre complex. Nature 1988, 336, 182–184. 10.1038/336182a0.

[ref53] Lin X.; Murchison H. A.; Nagarajan V.; Parson W. W.; Allen J. P.; Williams J. C. Specific alteration of the oxidation potential of the electron donor in reaction centers from Rhodobacter sphaeroides. Proc. Natl. Acad. Sci. U.S.A. 1994, 91, 10265–10269. 10.1073/pnas.91.22.10265. 7937938 PMC45000

[ref54] Wendel M.; Nizinski S.; Tuwalska D.; Starzak K.; Szot D.; Prukala D.; Sikorski M.; Wybraniec S.; Burdzinski G. Time-resolved spectroscopy of the singlet excited state of betanin in aqueous and alcoholic solutions. Phys. Chem. Chem. Phys. 2015, 17, 18152–18158. 10.1039/c5cp00684h. 26102081

[ref55] Snellenburg J. J.; Laptenok S.; Seger R.; Mullen K. M.; van Stokkum I. H. M. Glotaran: a java-based graphical user interface for the R package TIMP. J. Stat. Software 2012, 49, 1–22. 10.18637/jss.v049.i03.

[ref56] Jia Y.; Jonas D. M.; Joo T.; Nagasawa Y.; Lang M. J.; Fleming G. R. Observation of Ultrafast Energy Transfer from the Accessory Bacteriochlorophylls to the Special Pair in Photosynthetic Reaction Centers. J. Phys. Chem. 1995, 99, 6263–6266. 10.1021/j100017a001.

[ref57] Jonas D. M.; Lang M. J.; Nagasawa Y.; Joo T.; Fleming G. R. Pump–Probe Polarization Anisotropy Study of Femtosecond Energy Transfer within the Photosynthetic Reaction Center of Rhodobacter sphaeroides R26. J. Phys. Chem. 1996, 100, 12660–12673. 10.1021/jp960708t.

[ref58] Stanley R. J.; King B.; Boxer S. G. Excited State Energy Transfer Pathways in Photosynthetic Reaction Centers. 1. Structural Symmetry Effects. J. Phys. Chem. 1996, 100, 12052–12059. 10.1021/jp9614916.

[ref59] King B. A.; McAnaney T. B.; deWinter A.; Boxer S. G. Excited State Energy Transfer Pathways in Photosynthetic Reaction Centers. 3. Ultrafast Emission from the Monomeric Bacteriochlorophylls. J. Phys. Chem. B 2000, 104, 8895–8902. 10.1021/jp001745u.

[ref60] Jordanides X. J.; Scholes G. D.; Fleming G. R. The Mechanism of Energy Transfer in the Bacterial Photosynthetic Reaction Center. J. Phys. Chem. B 2001, 105, 1652–1669. 10.1021/jp003572e.

[ref61] van Brederode M. E.; Jones M. R.; van Mourik F.; van Stokkum I. H. M.; van Grondelle R. A New Pathway for Transmembrane Electron Transfer in Photosynthetic Reaction Centers ofRhodobacter sphaeroidesNot Involving the Excited Special Pair. Biochemistry 1997, 36, 6855–6861. 10.1021/bi9703756. 9188680

[ref62] van Brederode M. E.; van Mourik F.; van Stokkum I. H. M.; Jones M. R.; van Grondelle R. Multiple pathways for ultrafast transduction of light energy in the photosynthetic reaction center of Rhodobacter sphaeroides. Proc. Natl. Acad. Sci. U.S.A. 1999, 96, 2054–2059. 10.1073/pnas.96.5.2054. 10051593 PMC26735

[ref63] Fajer J.; Brune D. C.; Davis M. S.; Forman A.; Spaulding L. D. Primary charge separation in bacterial photosynthesis: Oxidized chlorophylls and reduced pheophytin. Proc. Natl. Acad. Sci. U.S.A. 1975, 72, 4956–4960. 10.1073/pnas.72.12.4956. 174084 PMC388853

[ref64] Peloquin J. M.; Williams J. C.; Lin X.; Alden R. G.; Taguchi A. K. W.; Allen J. P.; Woodbury N. W. Time-dependent thermodynamics during early electron transfer in reaction centers from Rhodobacter sphaeroides. Biochemistry 1994, 33, 8089–8100. 10.1021/bi00192a014. 8025115

[ref65] Lin S.; Taguchi A. K. W.; Woodbury N. W. Excitation Wavelength Dependence of Energy Transfer and Charge Separation in Reaction Centers fromRhodobacter sphaeroides: Evidence for Adiabatic Electron Transfer. J. Phys. Chem. 1996, 100, 17067–17078. 10.1021/jp961590j.

[ref66] Zhu J.; van Stokkum I. H. M.; Paparelli L.; Jones M. R.; Groot M. L. Early Bacteriopheophytin Reduction in Charge Separation in Reaction Centers of Rhodobacter sphaeroides. Biophys. J. 2013, 104, 2493–2502. 10.1016/j.bpj.2013.04.026. 23746522 PMC3672893

[ref67] Heller B. A.; Holten D.; Kirmaier C. Effects of Asp Residues Near the L-Side Pigments in Bacterial Reaction Centers. Biochemistry 1996, 35, 15418–15427. 10.1021/bi961362f. 8952494

[ref68] Katilius E.; Turanchik T.; Lin S.; Taguchi A. K. W.; Woodbury N. W. B-Side Electron Transfer in a Rhodobacter sphaeroides Reaction Center Mutant in Which the B-Side Monomer Bacteriochlorophyll Is Replaced with Bacteriopheophytin. J. Phys. Chem. B 1999, 103, 7386–7389. 10.1021/jp991670y.

[ref69] Dubas K.; Baranowski M.; Podhorodecki A.; Jones M. R.; Gibasiewicz K. Unified Model of Nanosecond Charge Recombination in Closed Reaction Centers from Rhodobacter sphaeroides: Role of Protein Polarization Dynamics. J. Phys. Chem. B 2016, 120, 4890–4896. 10.1021/acs.jpcb.6b01459. 27171418

