# Supplementary material for: Antagonistic Effects of Point Mutations on Charge Recombination and a New View of Primary Charge Separation in Photosynthetic Proteins
Source: J Phys Chem B. 2021 Jul 30;125(31):8742–56. doi: 10.1021/acs.jpcb.1c03978 (PMC8389993; doi:10.1021/acs.jpcb.1c03978)
Supplement: Supplementary file 1 — jp1c03978_si_001.pdf [file jp1c03978_si_001.pdf]

Supporting Information

Antagonistic Effects of Point Mutations on Charge Recombination and a New View of Primary Charge Separation in Photosynthetic Proteins

K. Dubas,[1, 3] , S. Szewczyk,[1] Rafał Białek [1], G. Burdziński,[1] M. R. Jones,[2] K. Gibasiewicz[1]*

[1] Faculty of Physics, Adam Mickiewicz University, ul. Uniwersytetu Poznanskiego 2, 61-614 Poznań, Poland.

[2] School of Biochemistry, Medical Sciences Building, University of Bristol, University Walk, Bristol, BS8 1TD, UK

[3] Department of Optometry, Poznan University of Medical Sciences, ul. Rokietnicka 5d, 60-806 Poznań, Poland

* Corresponding author; e-mail: krzyszgi@amu.edu.pl; tel: +48 61 8296390

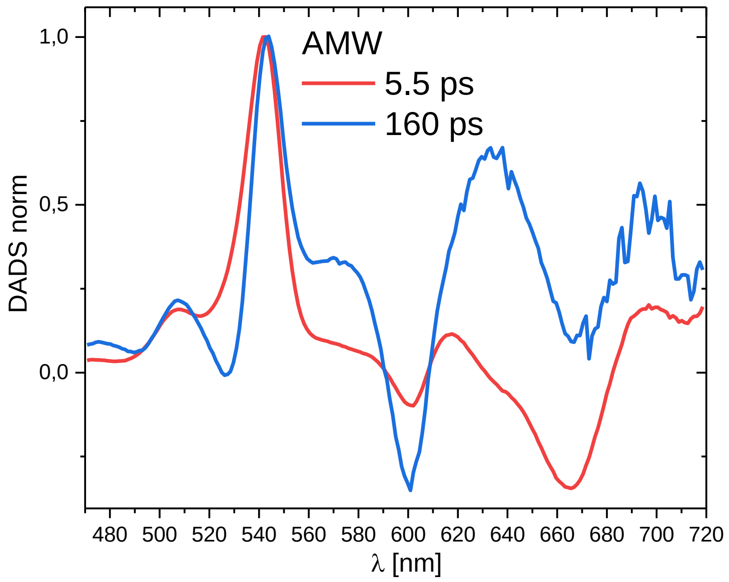

Figure S1. Comparison of the 5.5-ps and 160-ps DADS for the AMW RC normalized at ~542 nm.

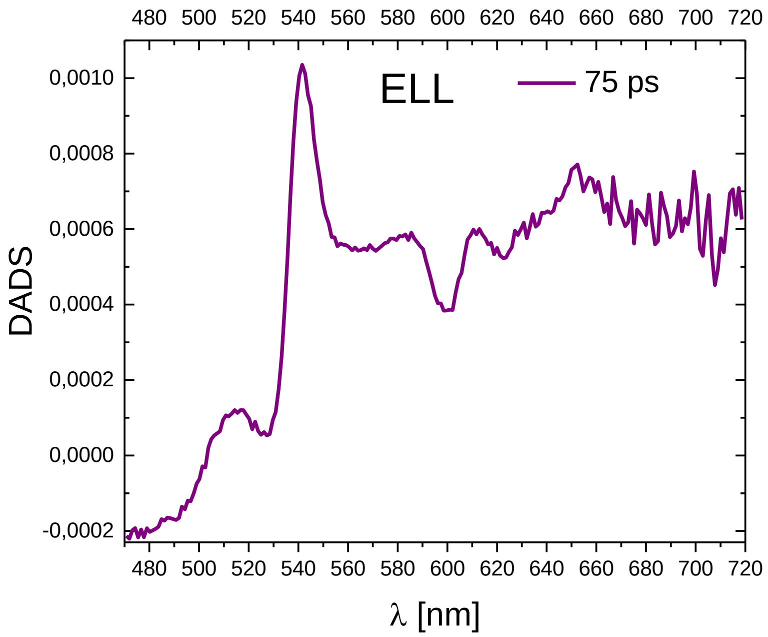

Figure S2. The 75-ps DADS of the ELL RC from Fig. 2C extended in the vertical direction.

Comparison of 6- and 7-exponential global fits for the ELL sample

The corresponding 570/590-ps DADS and 4.2/4.5-ns DADS for the ELL RC are of very similar shapes for the 6- and 7-exponential global fits (compare spectra in Figs. S3 and 2C). An extra component of ~75-ps lifetime in the 7-exponential fit caused shortening of the 3.2-ps and 17-ps components (resolved in the 6-exponential fit) to 2.2 and 5.7 ps, respectively (in the 7-exponential fit). Moreover, introduction of the ~75-ps DADS resulted in the shape and lifetime of the 2.2-ps DADS being very similar to those resolved for the three remaining RCs (Fig. 3A).

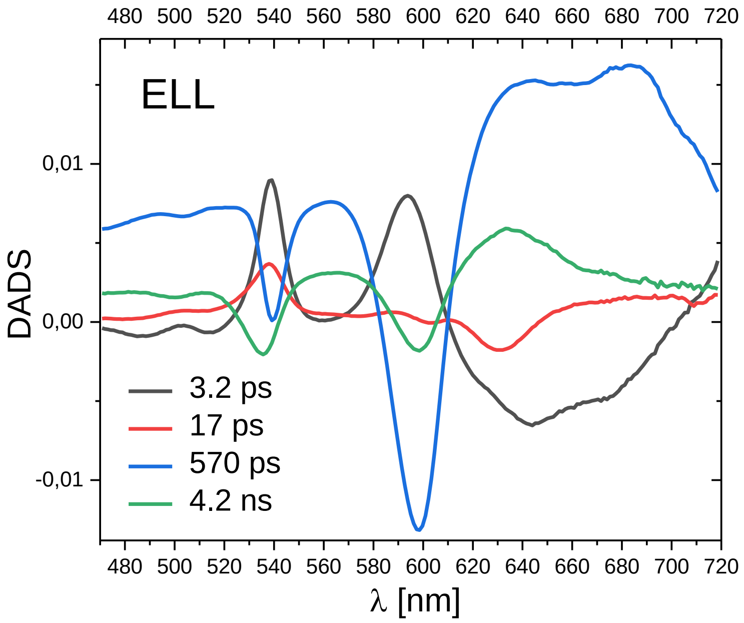

Figure S3. Decay associated difference spectra for the ELL RC resulting from the six-exponential model (the two fastest subpicosecond DADS are not shown) associated with the compartmental model shown in Fig. 5C.

Estimation of the relative contribution of the states $P^+B_A^-$ and $P^+H_A^-$ within the compartment "$(P^+H_A^-)_1$"

In the following we describe the way in which we estimated the relative contributions of the states $P^+B_A^-$ and $P^+H_A^-$ within the compartment "$(P^+H_A^-)_1$" in the ELL/AMW RC. Estimations for the remaining RCs were made in an analogous way.

We assumed that the $(P^+H_A^-)_2$ SADS in Fig. S4 represents pure $P^+H_A^-$ state. Consequently, we assumed that the negative band of this SADS at ~540 nm is exclusively due to photobleaching of the H_A Q_x band whereas the negative band at ~600 nm is exclusively due to photobleaching of the P Q_x band. From the relative amplitudes of these bands, ΔA_H and ΔA_P, using the Lambert-Beer law, we estimated the ratio of differential molar extinction coefficients of these two molecules at the respective wavelengths:

$$\Delta\varepsilon_{H,540}/\Delta\varepsilon_{P,600} = \Delta A_H/\Delta A_P = 1.17. \quad \text{(S1)}$$

The method for determining the amplitudes ΔA_H and ΔA_P shown in Fig. S4 is somewhat arbitrary but we cannot see any clear advantage of different methods to estimate these quantities.

Next we assumed that the "$(P^+H_A^-)_1$" SADS in Fig. S4 represents a mixture of the states $P^+H_A^-$ and $P^+B_A^-$, and that the differential molar extinction coefficient of B_A at ~600 nm, $\Delta\varepsilon_{B,600}$, is the same as that of P at ~600 nm and, for simplicity, assumed to be equal to 1 (arbitrary unit):

$$\Delta\varepsilon_{B,600} = \Delta\varepsilon_{P,600} = 1. \quad \text{(S2)}$$

We also assumed that the relaxation state of the protein does not affect the values of $\Delta\varepsilon_{H,540}$ and $\Delta\varepsilon_{P,600}$, meaning that they are the same for the compartments "$(P^+H_A^-)_1$" and $(P^+H_A^-)_2$.

Under these assumptions we could estimate the relative concentrations c_{PH} and c_{PB} (arbitrary unit) of the two states contributing to the "$(P^+H_A^-)_1$" compartment, $P^+H_A^-$ and $P^+B_A^-$, respectively, from the following set of linear equations obtained from the Lambert-Beer law:

- at ~540 nm

$$c_{PH}\,\Delta\varepsilon_{H,540} = \Delta A_H, \quad \text{(S3)}$$

- at ~600 nm

$$c_{PH}\,\Delta\varepsilon_P + c_{PB}\,(\Delta\varepsilon_B + \Delta\varepsilon_P) = \Delta A, \quad \text{(S4)}$$

where ΔA_H and ΔA are amplitudes of the ~540 and ~600-nm bands of the "$(P^+H_A^-)_1$" SADS, as shown in Fig. S4.

Finally, we estimated the free energy gap, ΔG_1, between the states $P^+H_A^-$ and $P^+B_A^-$ contributing to the compartment "$(P^+H_A^-)_1$":

$$\Delta G_1 = kT \ln(c_{PH}/c_{PB}), \quad \text{(S5)}$$

where k stands for a Boltzmann constant and T - an absolute temperature ($kT \approx 25$ meV).

In the case of WT sample, due to larger amplitude of "$(P^+H_A^-)_1$" SADS at ~540 nm than that of the $(P^+H_A^-)_2$ SADS, the latter one was multiplied by the arbitrarily selected correction factor of 1.3, the minimal value necessary to obtain consistent results.

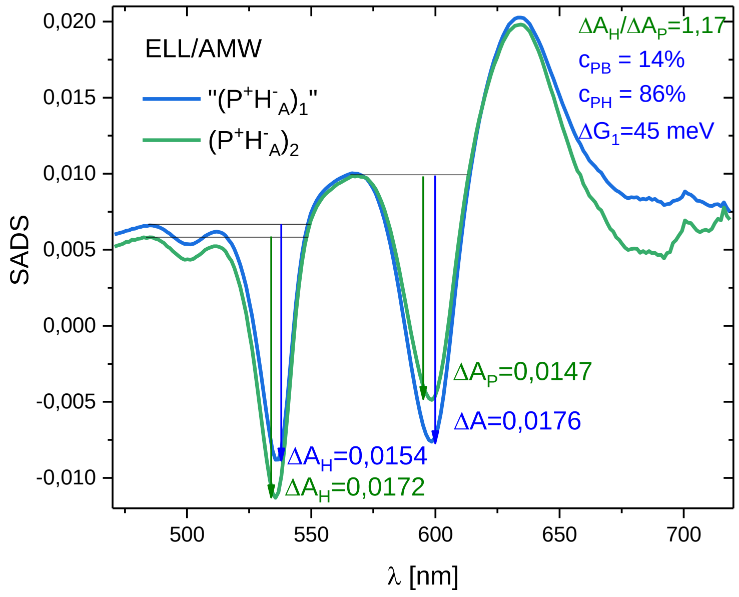

Figure S4. Graphical illustration on the determination of energetic parameters from the negative SADS bands amplitudes associated with the states "$(P^{+}H_{A}^{-})_{1}$" and $(P^{+}H_{A}^{-})_{2}$ of the ELL/AMW RC (spectra redrawn from Fig. 5D). ΔA_H – amplitude of the signal at ~540 nm ascribed to photobleaching of the H_A Q_x band; ΔA_P – amplitude of the signal at ~600 nm ascribed to photobleaching of the P Q_x band. See text for further details.

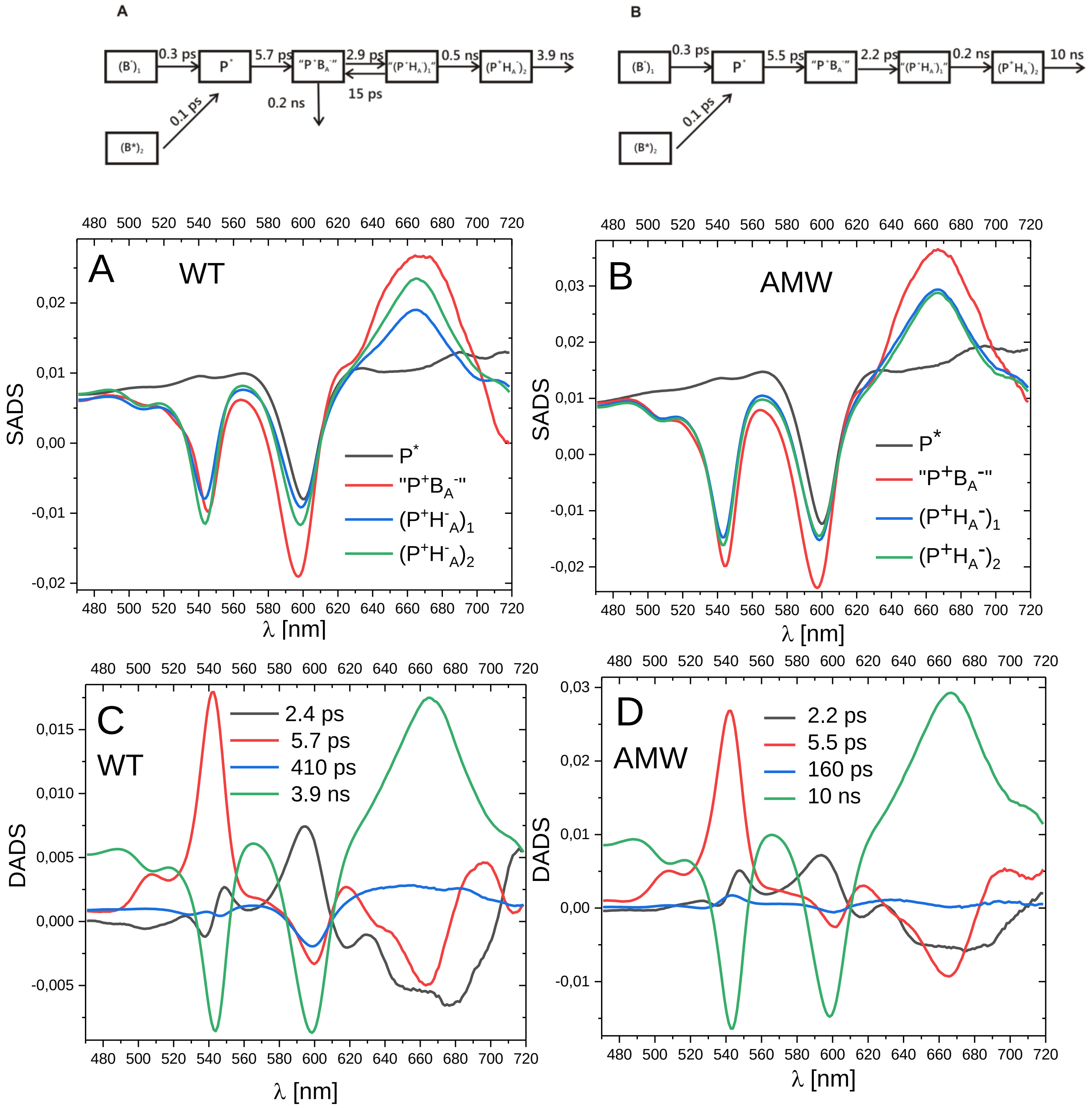

Figure S5. Results of target (A, B) and global (C, D) analysis for the WT and AMW RCs with molecular lifetime values τ_3 and τ_4 taken from Fig. 5A&B but exchanged and fixed. The remaining fit parameters were not fixed.
